# Design, Synthesis and Biological Evaluation of 7-Chloro-9*H*-pyrimido[4,5-*b*]indole-based Glycogen Synthase Kinase-3β Inhibitors

**DOI:** 10.3390/molecules24122331

**Published:** 2019-06-25

**Authors:** Stanislav Andreev, Tatu Pantsar, Francesco Ansideri, Mark Kudolo, Michael Forster, Dieter Schollmeyer, Stefan A. Laufer, Pierre Koch

**Affiliations:** 1 Institute of Pharmaceutical Sciences, Department of Medicinal and Pharmaceutical Chemistry, Eberhard Karls University Tübingen, Auf der Morgenstelle 8, 72076 Tübingen, Germany; stanislav.andreev@uni-tuebingen.de (S.A.); francesco.ansideri@uni-tuebingen.de (F.A.); Mark.Kudolo@uni-tuebingen.de (M.K.); Michael.forster@uni-tuebingen.de (M.F.); Stefan.laufer@uni-tuebingen.de (S.A.L.); 2 Department of Internal Medicine VIII, University Hospital Tübingen, Otfried-Müller-Str. 14, 72076 Tübingen, Germany; tatu.pantsar@uef.fi; 3 School of Pharmacy, University of Eastern Finland, P.O. Box 1627, 70211 Kuopio, Finland; 4 Department of Organic Chemistry, Johannes Gutenberg University Mainz, Duesbergweg 10-14, 55099 Mainz, Germany; scholli@uni-mainz.de; 5 Department of Pharmaceutical/Medicinal Chemistry II, Institute of Pharmacy, University of Regensburg, Universitätsstraße 31, 93053 Regensburg, Germany

**Keywords:** Glycogen synthase kinase-3β, 7-chloro-9*H*-pyrimido[4,5-*b*]indole, protein kinase, kinase inhibitor, tofacitinib

## Abstract

Glycogen synthase kinase-3β (GSK-3β) represents a relevant drug target for the treatment of neurodegenerative pathologies including Alzheimer’s disease. We herein report on the optimization of a novel class of GSK-3β inhibitors based on the tofacitinib-derived screen hit 3-((3*R*,4*R*)-3-((7-chloro-9*H*-pyrimido[4,5-*b*]indol-4-yl)(methyl)amino)-4-methylpiperidin-1-yl)-3-oxopropanenitrile (**1**). We synthesized a series of 19 novel 7-chloro-9*H*-pyrimido[4,5-*b*]indole-based derivatives and studied their structure–activity relationships with focus on the cyanoacetyl piperidine moiety. We unveiled the crucial role of the nitrile group and its importance for the activity of this compound series. A successful rigidization approach afforded 3-(3a*RS*,7a*SR*)-(1-(7-chloro-9*H*-pyrimido[4,5-*b*]indol-4-yl)octahydro-6*H*-pyrrolo[2,3-*c*]pyridin-6-yl)-propanenitrile (**24**), which displayed an IC_50_ value of 130 nM on GSK-3β and was further characterized by its metabolic stability. Finally, we disclosed the putative binding modes of the most potent inhibitors within the ATP binding site of GSK-3β by 1 µs molecular dynamics simulations.

## 1. Introduction

The human kinome represents a collective of over 500 genes dedicated to the sophisticated signaling networks in human cells [1]. With the majority of protein kinases being catalytically active, hence capable of transferring the γ-phosphate group from ATP/GTP to their protein substrates, this enzyme group is assumed to modify up to 30% of the intracellular proteome by phosphorylation [2]. While certain kinases target a limited number of substrates and consequently play a distinct role in cell signaling, others act as multitasking players in the cell circuitry. With over 30 proposed substrates glycogen synthase kinase-3 (GSK-3) clearly joins the ranks of the latter class [3]. Its name is attributed to the inhibition on glycogen synthesis within the frame of intracellular insulin signal transduction [4,5,6], yet not fully reflects the wide-ranging tasks of GSK-3 within the cell. The two isoforms of this serine/threonine kinase, namely GSK-3α and GSK-3β, differ in their C- and N-termini, while their kinase domains are highly conserved and characterized by 98% homology [7]. Despite their similarities, knock-out experiments in mice as well as numerous pharmacological studies have questioned the complete redundancy of these two paralogs [8,9,10,11].

GSK-3 is ubiquitously expressed and involved in at least five different signaling pathways (Insulin, Wnt, Notch, Hedgehog and TGF-β). This raises the question on how proper regulation mechanisms guarantee the flawless functionality of this enzyme [12]. A dysregulated GSK-3 activity has been linked to the development of several pathologies such as cancer, diabetes, CNS disorders, and neurodegeneration [11]. Thus, being a potential therapeutic target, GSK-3 has received attention of pharmaceutical research. Despite decades of extensive research efforts, several questions regarding the intracellular impact of this kinase, the mechanisms ensuring its regulation as well as its validity as a drug target remain unanswered. This has caused a strong demand for chemical agents, which can serve as chemical probes in pharmacological and biochemical studies and also as starting points for the development of clinical candidates.

**Scheme 1:**
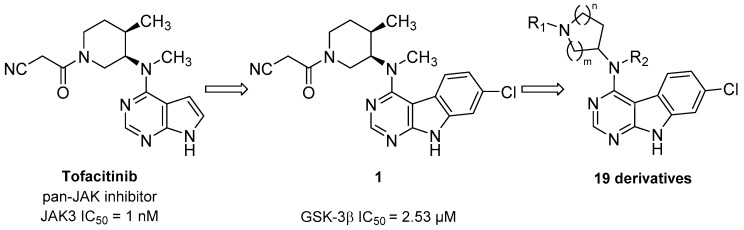
Development of glycogen synthase kinase-3β (GSK-3β) inhibitors derived from the pan-Janus kinase (JAK) inhibitor tofacitinib.

In this context, we herein present a novel class of 7-chloro-9*H*-pyrimido[4,5-*b*]indole-based GSK-3β inhibitors. The compound series is derived from the pan-Janus kinase (JAK) inhibitor tofacitinib (Scheme 1), formerly known as CP-690,550. The main target of tofacitinib is JAK3 [13]. This non-receptor tyrosine kinase is expressed exclusively in lymphoid cells, where it transduces the signals of γ-common cytokines in the course of the JAK-STAT signaling pathway [13,14]. A pharmacological intervention in this pathway with tofacitinib is used in the treatment of immunological disorders such as rheumatoid or psoriatic arthritis [15]. In the course of a kinome-wide screening campaign of our in-house compound library, the tofacitinib-derived 7-chloro-9*H*-pyrimido[4,5-*b*]indole **1** was identified as a GSK-3β inhibitor in the single-digit micromolar range (Scheme 1). Interestingly, GSK-3β and JAK3 are located in different branches of the phylogenetic kinome tree. They are structurally diverse, even their kinase domains display low homology (identity 22.6%) (Figure S1, Supplementary Materials).

In this work, we describe a medicinal chemistry strategy consisting of the design, synthetic preparation and biological evaluation of a series of compounds dedicated to an optimization of hit **1** towards GSK-3β inhibition. Interpretation of the biological data combined with in silico approaches provides insight into structure–activity relationships (SARs) and the putative binding mode of this novel class of GSK-3β inhibitors. Finally, the most promising inhibitors were further characterized by their metabolic stability in human liver microsomes (HLM) and inhibitory activity on JAK3, which is the main target of tofacitinib.

## 2. Results and Discussion

### 2.1. Biological Evaluation

**Figure 1:**
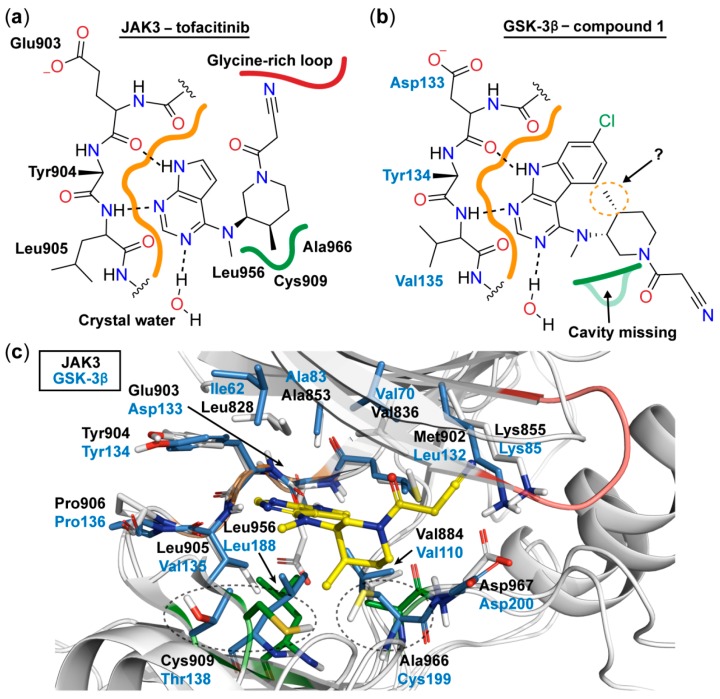
Schematic 2D representation of the binding mode of tofacitinib to JAK3 derived from its co-crystal structure (PDB ID: 3LXK) [16] (**a**) and the expected binding mode of **1** to GSK-3β (**b**). The lipophilic cavity is highlighted with green and the hinge region with orange. Hydrogen bonds are depicted as dashed lines. (**c**) 3D superposition based on the hinge regions of GSK-3β (PDB ID: 4PTC) and JAK3 (PDB ID: 3LXK) shows a high conservation of the residues in the ATP binding site [17]. The lipophilic cavity is missing in GSK-3β, as the spatial orientation of the Thr138 is totally different compared to the Cys909 in JAK3 and the residue Cys199 is bulkier than the Ala966 found in JAK3. Moreover, this Cys199 would clash with tofacitinib (yellow) in its JAK3 binding conformation. Residues near to tofacitinib are depicted as sticks. The stick coloring is the following: GSK-3β, blue; JAK3, grey; JAK3 residues forming the lipophilic cavity, green. The cartoon coloring is the following: hinge regions, orange; lipophilic cavity residues, green; JAK3 glycine-rich loop, red.

The binding mode of JAK3 in complex with tofacitinib (PDB ID: 3LXK) has been resolved (Figure 1) [16]. The 7*H*-pyrrolo[2,3-*d*]pyrimidine moiety of tofacitinib forms a bidentate hydrogen bond to the hinge residues Glu903 and Leu905, whereas the N-3 atom of the pyrimidine ring displays an interaction with a crystal water molecule. The ribose region of the kinase accommodates the piperidine ring of the inhibitor, allowing the exocyclic methyl group to occupy a miniscule lipophilic cavity in the bottom of the catalytic cleft formed by Ala966, Cys909, and Leu956. Consequently, this positioning of the methyl group leads to an orientation of the nitrile residue towards the glycine-rich loop.

Taking into account the high conservation of the ATP binding site, we expected a similar hydrogen bonding pattern to occur for the 7-chloro-9*H*-pyrimido[4,5-*b*]indole substructure in **1** with the hinge region of GSK-3β (Figure 1). Therefore, we decided to leave the chlorinated tricycle unmodified and set the focus of our optimization study on the aliphatic piperidine side chain. The distinct configuration of the two stereocenters within this moiety is a prerequisite for potent JAK3 inhibition and requires laborious steps in the enantiopure preparation of tofacitinib and hit **1**, respectively [18]. However, in contrast to JAK3, GSK-3β lacks a comparable lipophilic cavity in the bottom of its ATP binding site (Figure 1), which questioned the necessity of the corresponding exocyclic methyl group. This motivated us to prepare **14a** (Table 1). This compound, lacking the methyl substituent, has one stereocenter less and was prepared as racemic mixture. This approach simplified the synthetic preparation significantly. The racemic **14a** was found to exhibit a similar inhibitory activity on GSK-3β as **1**, justifying the selected approach, and was therefore chosen as a starting point for the development of further derivatives.

At first, we evaluated the importance of the carbonyl group within the cyanoacetamide moiety. Tertiary amine **14b**, which lacks the carbonyl group of **14a,** exhibited a 2.5-fold increase in activity. The molecular structures of **14b** and its amide counterpart display critical differences in their chemical properties. The loss of a possible hydrogen bond acceptor, a highly increased basicity of the piperidine nitrogen, as well as an enhanced flexibility of the cyanoethyl substituent provide putative explanations for the enhanced biological activity. With regard to this affinity shift into the nanomolar range this tertiary amine was assigned as the lead compound of this optimization study.

Next, we installed a methyl group on the indole nitrogen of **14b**. The resulting compound **14o**, uncapable of donating a hydrogen bond with its indole nitrogen, was inactive on GSK-3β. This negative outcome supported our hypothesis concerning the hydrogen bonding interactions of the 7-chloro-9*H*-pyrimido[4,5-*b*]indole scaffold to the hinge backbone of GSK-3β.

With this data in hand, our attention was refocused on the piperidine ring to assess the effects of the substituent **R_1_** (Table 1). In order to examine the chemical space around the cyanoethyl moiety of the lead compound **14b**, we prepared a series of diverse tertiary amines (**14c–n**). Our initial approaches consisted of the application of linear and branched lipophilic substituents such as propyl (**14e**), isopropyl (**14f**), cyclopentyl (**14g**), and cyclopropylmethyl (**14m**). The corresponding compounds displayed a severe decrease in activity on GSK-3β, which clearly indicates that aliphatic hydrocarbon substituents are not tolerated in this position. Within this inactive quartet, it is worth highlighting compound **14e**, whose propyl substituent maintains similar steric properties as in **14b**, however, **14e** lacks the nitrile group. Therefore, the significantly decreased activity of **14e** accentuates the importance of this functional group.

In order to disclose the vital role of the nitrile group for the activity of **14b**, we considered the following effects which can originate from its functionality. First, owing to the polarization of the triple bond, its nitrogen can serve as hydrogen bond acceptor. Such interactions have been observed in a number of nitrile-containing pharmaceuticals [19,20]. To evaluate this hypothesis, diverse substituents containing hydrogen bond acceptors, assigned to mimic the crucial nitrile group, were introduced on the piperidine nitrogen. These included both (hetero)aromatic rings like furane, pyridine or cyanobenzene in compounds **14h–j**, as well as carbonyl group-containing moieties (**14c** and **14d**). Surprisingly, all of these modifications were associated with a dramatic decrease in activity. Even compound **14n,** bearing a dimethylaminopropyl substituent, showed remarkably reduced GSK-3β inhibition.

At first glance, these findings might lead to the assumption of a hydrogen bond interaction being improbable for the nitrile group. However, the uniqueness of this functionality in terms of its rod-shaped geometry, resulting from the sp-hybridization of the carbon-nitrogen bond, must clearly be considered when interpreting these results. Most probably, the geometric and/or steric configuration of the other applied hydrogen bond-accepting substituents were not tolerated by the target enzyme.

Furthermore, taking into account that a nitrile group can significantly lower the electron density of atoms in its close proximity, we investigated the impact of the substituents on the basicity of the piperidine nitrogen. To this end, we calculated pK_a_ values for the piperidine nitrogen in compounds **14b–j** and **14m–n** (Table 1). The majority of pK_a_ values for this tertiary amine collective falls in the range between 6.8 and 8.3 predicting a significant degree of protonation of the nitrogen at pH 7.4. In contrast, **14b** was assigned with a significantly lower value of 5.5, which thoroughly reflects the electron withdrawing effects of the nitrile group and suggests a dominating presence of the uncharged free base under the physiological pH. This can be of great relevance for the binding of **14b** to GSK-3β. For instance, if the lone pair of the piperidine nitrogen is directed towards positively charged residues of the enzyme, a protonation would result in repulsion.

In this context, we hypothesized that the lowered pK_a_ and therefrom resulting unprotonated piperidine nitrogen might be an explanation for the significantly higher affinity of **14b**. To pursue this theory, we installed proper substituents on the piperidine nitrogen with the aim to lower the pK_a_ in a similar manner. A bioisosteric replacement of the nitrile with a highly electron withdrawing trifluoromethyl group afforded compound **14k**. In contrast, the nitrile group was maintained in compound **14l**, but is located closer to the piperidine nitrogen than in **14b**, amplifying its influence on the basicity. Even though the pK_a_ values of these molecules are in agreement with the original intentions (5.7 and 2.5, respectively), both derivatives were found to be inactive in the kinase assay. These negative results allowed us the conclusion that the protonation state of the piperidine nitrogen cannot solely determine the nanomolar activity of **14b**. Additionally, the drop in activity seen with **14l** points out that a specific distance between the nitrile group and the piperidine nitrogen is of utmost importance and once more emphasizes the cyanoethyl residue as preferred substituent in this position.

The outcomes with the above described set of derivatives encouraged us to keep the essential cyanoethyl moiety intact during the upcoming optimization steps. Taking into account that a proper position of the nitrile group was revealed a prerequisite for binding affinity, we attempted to fine-tune the orientation of the cyanoethyl substituent by modifications of the piperidine ring.

Alteration of the heterocyclic ring size to a pyrrolidine or azepane as well as relocation of the inner cyclic nitrogen within the piperidine delivered compounds **15**–**17**. These modifications resulted in marginal shifts in the orientation of the essential cyanoethyl substituent. However, the derivatives **15**–**17** did not conserve the nanomolar activity of the parent compound **14b**, which highlighted the original piperidine as the optimal scaffold within this series to properly orientate the cyanoethyl moiety. Therefore, we decided to return to this saturated heterocycle and considered rigidization approaches with the idea of stabilizing a bioactive conformation of this moiety. The bicyclic octahydro-1*H*-pyrrolo[2,3-*c*]pyridine side chain present in compound **24** was chosen for its chemical feasibility. This compound displayed an IC_50_ value of 130 nM, which equals a 6-fold increase in potency compared to its unrigidized counterpart **14b**. It is worth noticing, that the extended hydrocarbon scaffold of **24** results in an expanded lipophilic surface area of the molecule, which enables the possibility of its improved inhibition to be driven by entropic effects. However, this is not reflected by the calculated lipophilic ligand efficiency (cLLE) values of this series (Table 2). In turn, it appears plausible that the cyanoethyl substituent is forced into a favorable direction due to the restricted rotation of the piperidine ring.

### 2.2. Molecular Modelling

For the better understanding of the binding and interactions of our compounds to GSK-3β, we conducted 1 µs molecular dynamics (MD) simulations for the most potent compounds **14b** and **24** (for full movies and raw data see Supplementary Materials).

**Figure 2:**
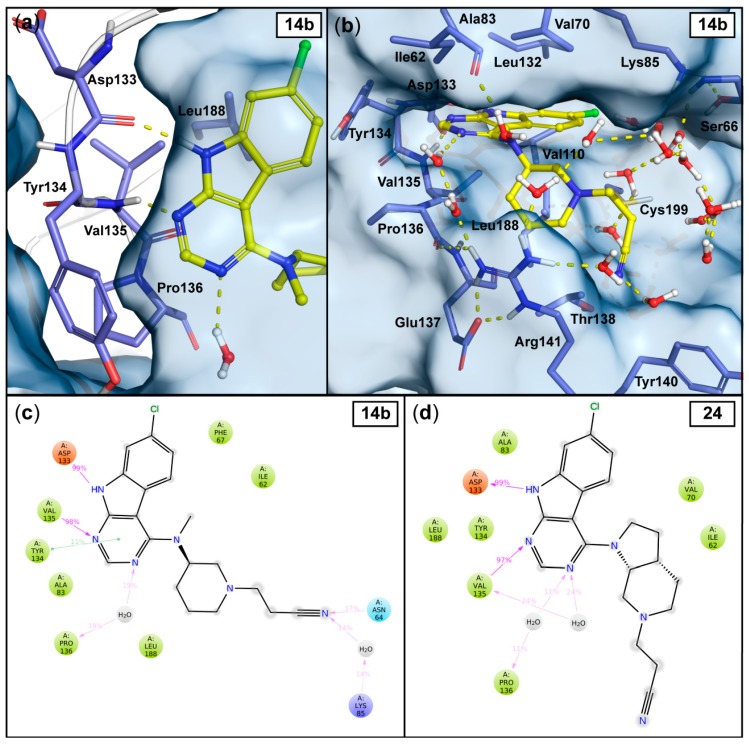
Observed interactions in the 1 μs MD simulations with the most potent compounds **14b** and **24**. The output conformation of compound **14b** (at 1000 ns) illustrates the stable hinge interactions with the Asp133 and Val135 and to the solvent (similar in **24**) (**a**), as well as the solvent exposure of the cyanoethyl moiety (**b**). Hydrogen bonds are depicted as yellow dashed lines and the water molecules within 4Å from the ligand are shown. The simulation interaction frequencies of compound **14b** (**c**) and compound **24** (**d**) were analyzed using the stabilized part of the simulations: 50–1000 ns and 30–1000 ns for **14b** and **24**, respectively (see Figure 3c–d). Interactions that appeared with more than 10% frequency are shown; hydrogen bonds are depicted as purple arrows, π–π interactions are depicted as green lines.

Throughout the simulations, the 7-chloro-9*H*-pyrimido[4,5-*b*]indole scaffold of both ligands exhibits stable interactions with the backbone of the hinge residues Asp133 and Val135 (Figure 2), while the halogen-substituted third aromatic ring of the scaffold is pointing towards the hydrophobic region I of the kinase.

**Figure 3:**
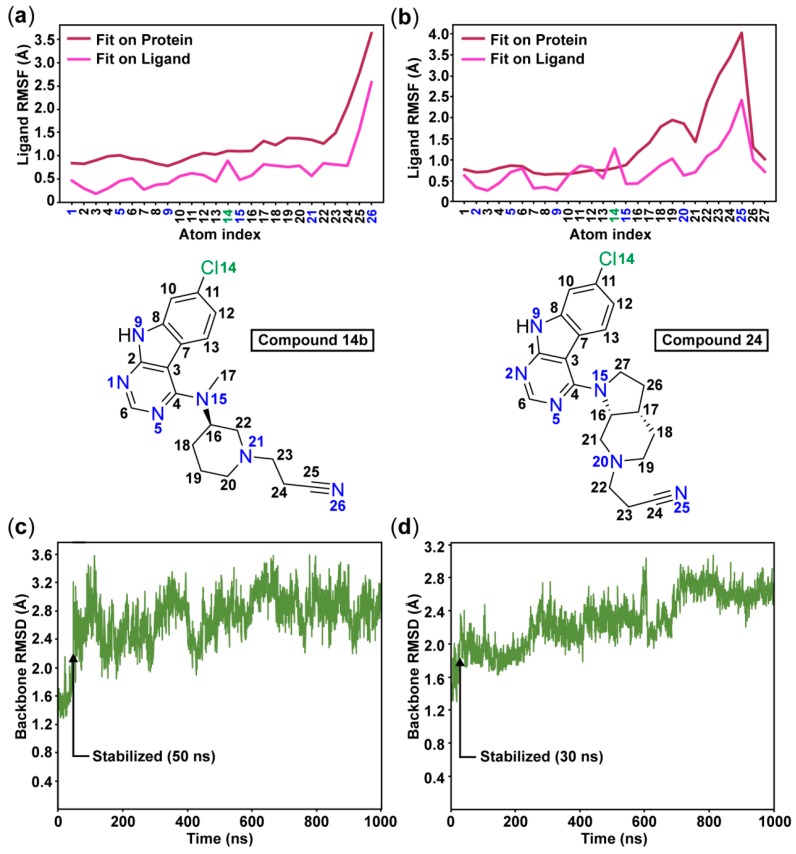
The root-mean-square fluctuations (RMSF) of the ligands **14b** (**a**) and **24** (**b**) illustrate the high flexibility of the cyanoethyl moieties throughout the simulations. The root-mean-square deviation (RMSD) of the protein shows that the simulations stabilize after 50 ns with compound **14b** (**c**) and after 30 ns with compound **24** (**d**).

In analogy to the observed crystal water interaction in the tofacitinib-JAK3 complex (see Figure 1), relatively stable water interactions were evident for the N-3 atom in the pyrimidine ring of our inhibitors (Figure 2). Additionally, a π–π interaction between this pyrimidine ring and the side chain of hinge residue Tyr134 appears in 11% frequency with **14b**. As expected, the sugar pocket is occupied by the piperidine heterocycle in case of **14b** or the octahydro-1*H*-pyrrolo[2,3-*c*]pyridine bicycle in case of **24**. While the hinge binding motifs of **14b** and **24** appear stable throughout the simulations, the cyanoethyl substituent is highly dynamic. These observations are well reflected by the ligand root-mean-square fluctuation (RMSF) for both ligands (Figure 3a–b). As far as the nitrile group is concerned, no single stable and specific interaction is observed. Rather a proper accommodation of the nitrile in the water interaction network is evident for both **14b** and **24**. A water-mediated interaction towards Lys85 as well as a direct hydrogen bond to Asn64 occurs in case of **14b**, however with relatively low frequencies (Figure 2c–d).

To disclose potential differences in the binding mode of the enantiomers of compound **24**, we conducted a 1 µs MD simulation also for the other enantiomer of **24** (3a*R*, 7a*S*). As comparable behavior between the enantiomers was observed (Figure S2, Supplementary Materials), we expect a similar binding mode to occur with compound **24** regardless of the enantiomer.

Overall, examples of nitrile groups functioning as hydrogen bond acceptors in ligand–protein complexes are described in literature including well-characterized examples of direct contacts to asparagine residues, as well as interactions mediated through water bridges [19,20]. Therefore, the interactions observed in the simulations appear plausible despite their low frequencies. Finally, although we used the most recent state-of-the-art force field for drug-like small molecules, OPLS3e [24], a potential inaccuracy in the characterization of the cyanoethyl group by the force field needs to be considered (e.g., lack of polarization).

### 2.3. ATP competition, JAK3 Inhibition, and Metabolic Stability

To extend the biological profile of this novel class of 7-chloro-9*H*-pyrimido[4,5-*b*]indole-based GSK-3β inhibitors, we tested the lead compound **14b** for its ATP competition in our ADP Glo assay system. An incubation of **14b** with an increased concentration of ATP (100 µM and 500 µM) resulted in a reduced inhibition potential for GSK-3β. The corresponding IC_50_ values were determined as 2.684 µM and 9.260 µM, respectively (Table S1 and Figure S3, Supplementary Materials). This suggests an ATP-competitive type of inhibition, which supports our theory of a binding mode within the ATP site of the kinase.

Furthermore, **14b** and **24** were examined for their inhibitory activity on the possible off-target JAK3, as this class of GSK-3β inhibitors is derived from the pan-JAK inhibitor tofacitinib. However, both compounds displayed no significant inhibition of JAK3 in an enzyme-linked immunosorbent assay (Table S2, Supplementary Materials) [25].

In a HLM stability assay, **14b** and **24** were intensively degraded during a timeframe of 120 min (25% and 8% remaining parent compound at the endpoint of the experiment, respectively; for details see Tables S3 and S4 as well as Figures S4 and S5, Supplementary Materials). The corresponding unsubstituted piperidines were observed as prominent metabolites.

### 2.4. Chemistry

**Scheme 2:**
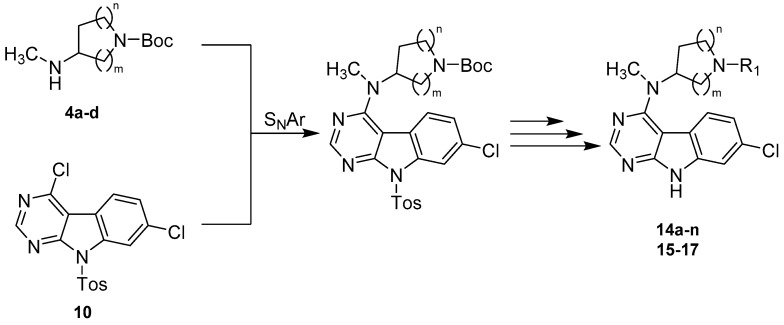
Synthetic strategy towards 7-chloro-9*H*-pyrimido[4,5-*b*]indole-based GSK-3β inhibitors.

Our synthetic strategy to access the majority of the herein presented 7-chloro-9*H*-pyrimido[4,5-*b*]indole-based GSK-3β inhibitors featured a convergent approach consisting of the preparation of the key intermediate 4,7-dichloro-9-tosyl-9*H*-pyrimido[4,5-*b*]indole (**10**) as well as the separate synthesis of appropriate alicyclic amine side chains (**4a–d**) (Scheme 2). Both building blocks were then fused via a nucleophilic aromatic substitution (S_N_Ar), with successive deprotection and derivatization procedures to afford the final compounds **14a–n** and **15–17**.

**Scheme 3:**
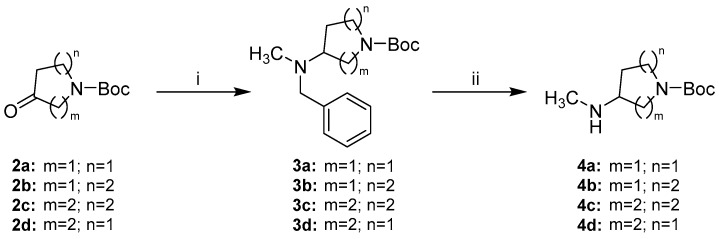
Synthetic route to alicyclic secondary amine side chains **4a–d**. Reagents and conditions: (**i**) *N*,*N*-benzylmethylamine, Na(OAc)_3_BH, AcOH, DCM, rt, (69–86%); (**ii**) H_2_ (5 bar), Pd/C, MeOH or EtOAc/MeOH 3:2, rt in case of **4a–4c** or Pd/C and Pd(OH)_2_/C, MeOH, rt in case of **4d**, (96–98%).

The preparation of alicyclic secondary amine side chains **4a**–**d** was achieved in two steps via benzyl protected amines **3a–d** adapting the strategy of Le Bourdonnec et al. (Scheme 3) [26]. The commercially available *N*-Boc protected cyclic ketones **2a**–**d** were reductively aminated with *N*,*N*-benzylmethylamine to give tertiary amines **3a**–**d** in moderate to high yields. Sodium triacetoxyborohydride was used as reductive agent facilitating a convenient one-pot procedure. Hydrogenolytic benzyl cleavage with a palladium catalyst gave secondary amines **4a**–**d** in nearly quantitative yields.

**Scheme 4:**
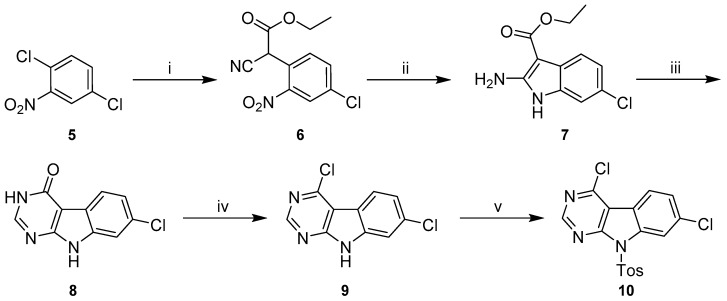
Synthetic route to 4,7-dichloro-9-tosyl-9*H*-pyrimido[4,5-*b*]indole (**10**). Reagents and conditions: (**i**) ethyl-2-cyanoacetate, NaH, DMF, 0 °C to 80 °C, (quant.); (**ii**) Zn, AcOH, 90 °C, (93%); (**iii**) NH_4_HCO_2_, formamide, 160 °C, (86%); (**iv**) POCl_3_, chlorobenzene, rt to 80 °C, (51%); (**v**) p-toluenesulfonyl chloride, NaH, THF, rt, (99%).

Synthetic literature provides numerous examples for the preparation of 9*H*-pyrimido[4,5-*b*]indoles with varying substituents on the non-heteroaromatic ring [27,28,29,30,31]. These typically include four-step procedures starting from appropriately decorated o-halonitrobenzenes, as the substitution pattern of the non-heteroaromatic ring is defined by this starting material. Modifying the protocols published by Reader et al. [31], we prepared key intermediate 4,7-dichloro-9-tosyl-9*H*-pyrimido[4,5-*b*]indole (**10**) starting from 1,4-dichloro-2-nitrobenzene (**5**) (Scheme 4).

In the initial step the commercially available **5** underwent an S_N_Ar with the sodium salt of ethyl-2-cyanoacetate, generated in situ with sodium hydride. The resulting intermediate **6** was then subjected to reductive conditions using elemental zinc in acetic acid. The crude product obtained in this cyclization reaction was a mixture of two compounds, which we assume to be the desired 2-aminoindole **7** and the corresponding 1-hydroxy derivative, in analogy to some closely related structures described by Showalter et al. [27]. Treatment of this mixture with formamide under forcing conditions resulted predominantly in **8**, whose carbonyl function was chlorinated with stochiometric amounts of POCl_3_ in chlorobenzene following the methodology described by Arnott et al. [32].

**Scheme 5:**
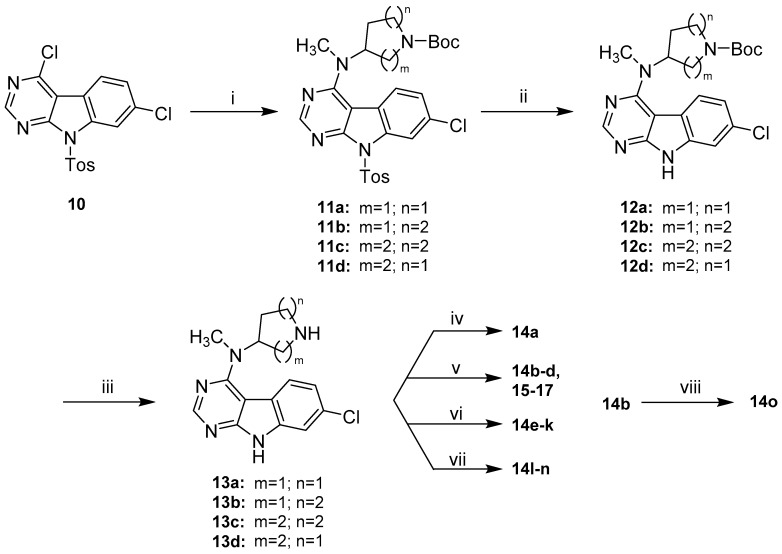
Synthetic route to final compounds **14a–o** listed with their structures in Table 1 and **15–17** listed with their structures in Table 2. Reagents and conditions: (**i**) **4a–d**, DIPEA, DMF, 80 °C, (93% to quant.); (**ii**) K*t*BuO, THF, rt, (65–78%); (**iii**) TFA, DCM, rt, (74–99%); (**iv**) **13b**, cyanoacetic acid, PyBOP, DIPEA, DCM, rt, (59%); (**v**) **13a–d**, acrylonitrile, MeOH, rt in case of **14b** and **15–17** or **13b**, corresponding acrylic acid derivative, MeOH, rt in case of **14c–d**, (63–89%); (**vi**) **13b**, corresponding aldehyde or ketone, AcOH, Na(OAc)_3_BH, DCM, rt, (43–74%); (**vii**) **13b**, bromoacetonitrile, Et_3_N, DMF, rt in case of **14l** or **13b**, (bromomethyl)cyclopropane, Et_3_N, MeCN, 60 °C in case of **14m** or **13b**, 3-chloro-*N*,*N*-dimethylpropan-1-amine ·HCl, Et_3_N, MeCN, 90 °C in case of **14n**, (39–91%); (**viii**) methyl iodide, NaH, THF, rt, (60%).

First attempts of a direct reaction of **9** with the alicyclic amine side chains gave unsatisfactory results, in the form of either prolonged reaction times or significant by-product formation, owing to harsh thermal conditions. The installation of a toluenesulfonyl (Tos) protecting group on the indole nitrogen in **10** significantly enhanced the reactivity of the pyrimidine ring and therefore allowed a clean nucleophilic substitution under relatively mild conditions in DMF and presence of DIPEA (Scheme 5). The sulfonamide protecting group was removed subsequently under basic conditions. A modification of the protocol described by Xu et al. using a high excess of K*t*BuO (7 eq.) instead of refluxing conditions allowed a fast deprotection at room temperature [33]. This approach gave intermediates **12a**–**d** in satisfying purity and yields (64–77% over three steps), justifying the synthetic detour associated with the protection of the indole nitrogen. **12a**–**d** were treated with TFA in DCM to convert them into the corresponding secondary amines **13a**–**d [34]**. These intermediates were then properly decorated by amide coupling, reductive amination [34], nucleophilic substitution [35] or Michael-addition [36] to give final compounds **14a**–**n** and **15**–**17** listed in Table 1 and Table 2, respectively. For the synthesis of **14o**, lead structure **14b** was methylated on its indole nitrogen applying a common procedure.

**Scheme 6:**
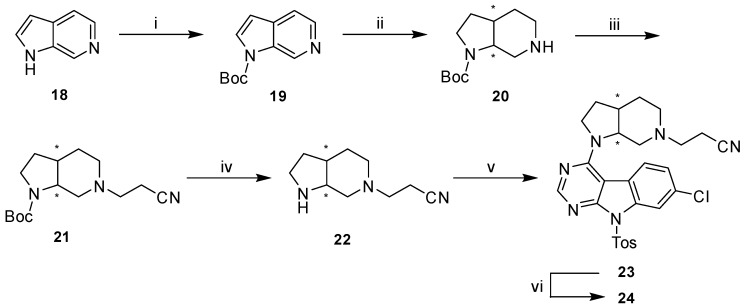
Synthetic route to final compound **24**. Reagents and conditions: (**i**) Boc_2_O, THF, 0 °C to rt, (92%); (**ii**) H_2_ (5 bar), PtO_2_, AcOH, rt, (88%); (**iii**) acrylonitrile, MeOH, rt, (83%); (**iv**) 4N HCl in dioxane, DCM, rt, (83%); (**v**) **10**, DIPEA, DMF, 80 °C, (64%); (**vi**) K*t*BuO, THF, rt, (55%).

For the preparation of the rigidized precursor **22** (Scheme 6) we followed a different synthetic approach compared to the aforementioned secondary amines **4a–d**. The use of 6-azaindole (**18**) as starting material allowed a selective, high-yielding protection of the pyrrole ring nitrogen with Boc anhydride in THF. The key step of this synthetic route was the following hydrogenation which we achieved under comparably mild conditions using Adam’s catalyst in glacial acetic acid giving the free piperidine base **20 [37]**.

In order to decrease the number of subsequent steps the crucial cyanoethyl moiety was directly installed on the piperidine nitrogen rather than an orthogonal protecting group. This conversion was readily achieved by Michael-addition with acrylonitrile in MeOH (**21**). Finally, acidic conditions were applied to cleave the protecting carbamate yielding **22 [38]**, which was reacted with **10** under similar S_N_Ar conditions with subsequent basic detosylation to afford final compound **24**.

**Figure 4:**
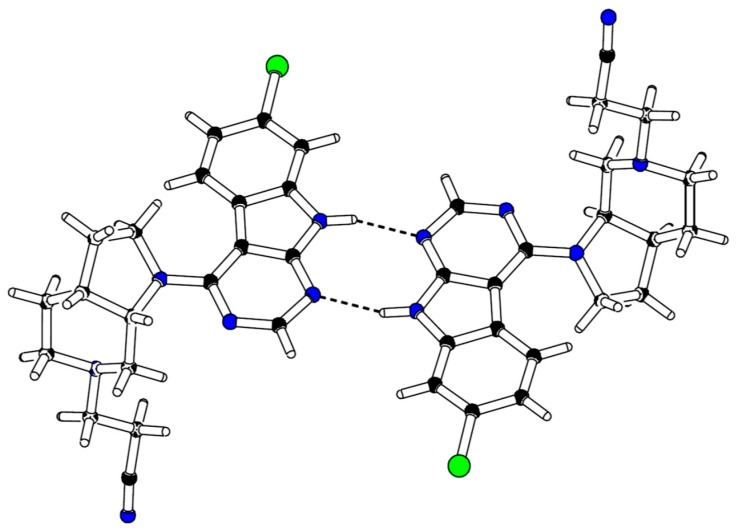
X-ray crystal structure of compound **24**.

In order to define the stereochemistry at the two stereocenters of the octahydro-1*H*-pyrrolo[2,3-*c*]pyridine ring, which were generated during the hydrogenation of Boc-protected azaindole **19**, single crystal analysis of **24** was performed (for details, see Table S5, Supplementary Materials). As expected, X-ray data confirmed that solely a mixture of the (3a*R*, 7a*S*) and (3a*S*, 7a*R*) enantiomers of **24** was obtained (Figure 4).

## 3. Conclusions

Starting from the micromolar GSK-3β screen hit **1**, we prepared a series of tofacitinib-derived 7-chloro-9*H*-pyrimido-[4,5-*b*]indole-based compounds. We characterized these compounds in their biological activity in an ADP Glo GSK-3β assay. A switch from amides to tertiary amines proved beneficial for the binding affinity regarding the substituent on the piperidine nitrogen. Moreover, a cyanoethyl substituent revealed to be an essential structural feature within this series. We examined the role of the nitrile group in detail and assume that it undergoes crucial hydrogen bonding interactions. We derived a putative binding mode for the most potent inhibitors **14b** and **24** from 1 µs molecular dynamics simulations. Most importantly, these inhibitors displayed no significant inhibition of the off-target JAK3. However, we observed a lack of metabolic stability in our HLM experiments, which is currently addressed in further optimization studies regarding this novel class of GSK-3β inhibitors.

## 4. Materials and Methods

### 4.1. Comparison of JAK3 and GSK-3β

The location of JAK3 and GSK3β in the phylogenetic tree of the human kinome was visualized by KinMap [39]. The sequence alignment was conducted to annotated kinase domains of the Uniprot entries P52333 (JAK3) and P49841 (GSK3B) with Clustal Omega (1.2.4) [40,41,42].

### 4.2. Molecular Modelling

All the modelling was conducted with Maestro Small-Molecule Drug Discovery Suite 2019-1 (Schrödinger, LLC, New York, NY, USA). The figures and the supplementary movies were prepared with PyMOL 2.2.3 (Schrödinger, LLC).

#### 4.2.1. pK_a_ Calculations

We used the Jaguar pK_a_ module (Schrödinger, LLC) for the pK_a_ predictions [43,44,45]. The calculations were conducted with default settings, except the conformational search step was omitted, as for the input conformation we used the lowest energy structure obtained from QM Conformer & Tautomer Predictor tool (Schrödinger, LLC). In brief, the QM Conformer & Tautomer Predictor workflow is the following. First, the proton donor and acceptor atoms are identified, and protons are redistributed among these to form a list of tautomers (protons can also be added to or subtracted from the input molecule). The generated tautomers were next ranked by their semiempirical PM3 heat of formation, and the high-energy tautomers were then discarded. For the surviving tautomers, a set of conformers were generated with MacroModel and the high-energy structures were eliminated by their semiempirical PM3 heat of formation. Subsequently, DFT geometry optimizations were performed on the surviving structures, using the B3LYP-D3/LACVP** level of theory. Finally, the structures were ranked using single-point energies at the M06-2X/cc-pVTZ(-f) calculated at the optimal geometries from the previous step.

#### 4.2.2. MD Simulations

For the simulations, we used the GSK-3β crystal structure PDB ID: 4PTC [17]. First, the structure (protein–co-crystallized ligand) was prepared by Protein Preparation Wizard [46] (default settings) using OPLS3e force field [24]. Next, the co-crystallized ligand was replaced by the compound 14b or 24, using the QM Conformer & Tautomer Predictor (for details see 4.2.1) output conformation of the compounds (second lowest energy structure for 14b, Boltzmann population: 20.0%; lowest energy structure for 24, Boltzmann population: 15.6%; lowest energy structure for 24 (3a*R*, 7a*S*), Boltzmann population: 13.0%). The MD simulations were conducted with Desmond [47] using OPLS3e force field [24], except the charges derived from the QM Conformer & Tautomer Predictor were applied for the ligands. The systems were solvated in a cubic box (edges 13 Å from the protein) and neutralized with counterions (Cl^–^) with 0.15 M KCl salt. The water was described with TIP3P water model [48]. The final systems consisted of 67,285 (14b) and 71,836 (24, both enantiomers) atoms. Prior to the 1000 ns production simulations, the default relaxation protocol of Desmond was conducted. The final simulations were conducted in NPT ensemble (310 K, thermostat: Nosé-Hoover chain; 1.01325 bar, barostat: Martyna–Tobias–Klein) with the default timestep of 2 fs and cut-off radius of 9.0 Å for Coulombic interactions.

### 4.3. Chemistry

#### 4.3.1. General Information

All solvents and reagents were commercially obtained and used without additional purification. High performance liquid chromatography (HPLC) was performed on an Hewlett Packard HP1090 series II HPLC system (Hewlett-Packard, Palo Alto, CA, USA) or an Agilent 1100 series HPLC system (Agilent, Santa Clara, CA, USA) equipped with a Phenomenex Luna 5 µm C8(2) 100 Å RP column (150 × 4.6 mm) (Phenomenex, Torrance, CA, USA) and a diode array detector detecting at 230 nm and 254 nm. The method consisted of elution using mobile phase A (MeOH) and mobile phase B (aqueous 0.01M KH_2_PO_4_ buffer, pH 2.3) in a flow of 1.5 mL/min and the gradient as follows: mobile phase A 40% to 85% during 8 min, mobile phase A 85% constant for 5 min, mobile phase A 85% to 40% during 1 min, mobile phase A 40% constant for 2 min; complete run time 16 min; injection volume 5 µL. Purity of final compounds was determined at 254 nm. Gas chromatography-mass spectrometry (GC-MS) was performed on a Hewlett-Packard HP 6890 Series GC System equipped with an HP 5973 mass selective detector (electron impact ionization) (Hewlett-Packard, Palo Alto, CA, USA). Helium 6.0 was used as carrier gas with a flow of 1.2 mL/min. In *method A* a Zebron ZB-5 column (30 m × 0.25 mm; 0.25 µm film thickness) (Phenomenex, Torrance, CA, USA) was used with the following temperature gradient: hold 160 °C for 1 min, from 160 °C to 240 °C during 8 min, hold 240 °C for 3 min, from 240 °C to 270 °C during 3 min, hold 270 °C for 3 min, from 270 °C to 300 °C during 3 min, hold 300 °C for 12 min; complete run time 33 min. In *method B* an Agilent J&W DB-5ms (30 m × 0.25 mm; 0.25 µm film thickness) (Agilent, Santa Clara, CA, USA) was used with the following temperature gradient: hold 100 °C for 5 min, from 100 °C to 320 °C during 22 min, hold 320 °C for 5 min; complete run time 32 min. Electrospray ionization mass spectrometry (ESI-MS) was performed on an Advion expression^s^ CMS TLC-ESI-MS coupling system (Advion, Ithaca, NY, USA) operating in ESI+ mode (capillary temperature 250 °C, capillary voltage 180V, source gas temperature 250 °C, ESI voltage 3500V) and ESI-mode (capillary temperature 250 °C, capillary voltage 180V, source gas temperature 250 °C, ESI voltage 2500V), elution with MeOH. Flash column chromatography was performed on an Interchim puriflash 430 or XS 420 (Interchim, Montluçon, France) on Grace Davison Discovery Sciences Davisil Chromatographic Silica Media LC60A (20–45 µm) (Grace Davison Discovery Sciences, MD, USA) or Interchim puriflash prepacked silica columns (SIHP-JP, 30 µm) (Interchim, Montluçon, France) and Merck Geduran Si60 63–200 µm silica gel (Merck, Darmstadt, Germany) for pre-columns. Mobile phases are described in the detailed procedures. Nuclear magnetic resonance (NMR) analysis was performed on 200, 300, and 400 MHz Bruker Avance spectrometers (Bruker, Billerica, MA, USA). Spectra were calibrated to residual peaks of utilized solvents, chemical shifts are reported in parts per million (ppm) relative to tetramethylsilane (δ = 0). Compounds with amide substituents (Boc protected intermediates and compounds **1** and **14a**) often displayed mixtures of amide bond rotamers in their NMR spectra. Thin layer chromatography (TLC) was performed on silica gel coated aluminum sheets (Merck TLC Silica gel F_254_, Merck, Darmstadt, Germany or Macherey-Nagel Alugram Sil G/UV_254_, Macherey-Nagel, Düren, Germany) with visualization under UV light at 254 nm or by ninhydrin stain.

#### 4.3.2. General Procedures

##### General Procedure A for the Preparation of Intermediates **3a–d** by Reductive Amination

The corresponding cyclic ketone (**2a**–**d**) (1 eq.) was dissolved in dry DCM. Glacial AcOH (1.1–1.15 eq.) and *N*,*N*-benzylmethylamine (1.1–1.25 eq.) were added, followed by portion-wise addition of Na(OAc)_3_BH (1.5–1.6 eq.). The mixture was stirred at rt, under N_2_ atmosphere and over Na_2_SO_4_ (0.5–1 g) overnight. In case of incomplete consumption of the ketone, additional amine and reductive agent were added to the mixture. After complete conversion, saturated NaHCO_3_ solution was added and phases were separated. The aqueous layer was extracted thrice with DCM. Combined organic layers were dried over Na_2_SO_4_ and concentrated under reduced pressure. The residue was purified by flash column chromatography.

##### General Procedure B for the Preparation of Intermediates **11a–d** by S_N_Ar

The appropriate secondary amine (**4a**–**d**) (1.25–1.4 eq.) and DIPEA (3 eq.) were added to a suspension of **10** (1 eq.) in dry DMF. The mixture was stirred at 70–80 °C overnight and poured into ice-cold water after completion. Saturated NH_4_Cl solution was added and the formed precipitate filtered, washed with cold water and dried over P_2_O_5_ in vacuo.

##### General Procedure C for the Preparation of Intermediates **12a–d** by Deprotection of Tosyl Protecting Group

The corresponding intermediate (**11a**–**d**) (1 eq.) was dissolved in THF (dry or HPLC grade) and K*t*BuO (7 eq.) was added. The mixture was stirred at rt and under N_2_ atmosphere for 0.5 h to 2 h. Saturated NH_4_Cl solution was added and the aqueous phase extracted three to four times with EtOAc. Combined organic layers were dried over Na_2_SO_4_. Volatiles were removed under reduced pressure and the residue purified by flash column chromatography.

##### General Procedure D for the Preparation of Intermediates **13a–d** by Deprotection of Boc Protecting Group

The appropriate Boc protected amine (**12a**–**d**) was dissolved or suspended in dry DCM and stirred. Subsequently trifluoroacetic acid (TFA) was added resulting in a 17% (*V/V*) solution which was stirred at rt for 0.5 h to 1.5 h. Volatiles were removed under reduced pressure and the highly acidic residue neutralized by addition of saturated NaHCO_3_ solution (30 mL). The high polarity of products required repetitive extraction with EtOAc and addition of MeOH to improve solubility. Combined organic layers were washed thrice with saturated NaHCO_3_ solution and dried over Na_2_SO_4_. Volatiles were removed under reduced pressure and the residue purified by flash column chromatography or directly used in the next step.

##### General Procedure E for the Preparation of Final Compounds **14e–k** by Reductive Amination

Intermediate **13b** (1 eq.), glacial AcOH (2 eq.) and the corresponding aldehyde or ketone (1.2–40 eq.) were stirred in dry DCM. Na(OAc)_3_BH (1.5–2.0 eq.) was added and the reaction stirred at rt, under N_2_ atmosphere and over some Na_2_SO_4_ (~ 50 mg) for 2 h to 6 h. When reaction control by HPLC indicated sufficient conversion, the mixture was diluted with DCM, washed four times with saturated NaHCO_3_ solution, dried over Na_2_SO_4_ and concentrated under reduced pressure. The residue was purified by flash column chromatography.

#### 4.3.3. Detailed Procedures

##### Preparation of **1**

3-((3*R*,4*R*)-3-((7-Chloro-9*H*-pyrimido[4,5-*b*]indol-4-yl)(methyl)amino)-4-methylpiperidin-1-yl)-3-oxopropanenitrile (**1**). 4,7-Dichloro-9*H*-pyrimido[4,5-*b*]indole (**9**) (50.0 mg, 0.21 mmol) and 3-((3*R*,4*R*)-4-methyl-3-(methylamino)piperidin-1-yl)-3-oxopropanenitrile hydrochloride [49] (48.7 mg, 0.21 mmol) were suspended in a mixture of dry dioxane (1 mL) and dry DMF (0.1 mL). DIPEA (67.9 mg, 0.53 mmol) was added and the mixture stirred under microwave irradiation (120 °C, 130W) in a sealed tube for 26 h. Volatiles were removed under reduced pressure. Purification of the residue by flash column chromatography (SiO_2_, 1.EtOAc:*i*PrOH 8:1, 2.DCM:MeOH 95:5) gave 24 mg of a light brown solid (29% yield); NMR shows a 3:1 mixture of amide bond rotamers, ^1^H-NMR (400 MHz, acetone-*d_6_*) δ 11.30 (s, 1H), 8.55–8.38 (m, 1H), 7.81 (d, *J* = 8.5 Hz, 1H), 7.62 (s, 1H), 7.33 (d, *J* = 8.4 Hz, 1H), 4.85–4.74 (m, 0.75H), 4.27–4.17 (m, 0.25H), 4.11–3.64 (m, 6H), 3.19 (s, 2.25H), 3.05 (s, 0.75H), 2.51–2.31 (m, 1H), 2.01–1.79 (m, 2H), 1.24 (d, *J* = 6.8 Hz, 0.75H), 1.15 (d, *J* = 6.9 Hz, 2.25H); ^13^C-NMR (101 MHz, acetone-*d_6_*) δ 164.5, 164.0, 161.0, 158.8, 155.3, 138.7, 131.4, 124.5, 122.0, 119.6, 116.0, 112.2, 100.0, 56.1, 53.3, 48.4, 46.6, 46.2, 34.1, 34.0, 32.9, 32.5, 32.2, 26.0, 25.7, 14.9; ESI-MS: (*m/z*) 396.9 [M + H]^+^, 418.9 [M + Na]^+^, 394.9 [M − H]^-^; HPLC: t_r_ = 7.201 min (100.0% purity).

##### Detailed Procedures for the Preparation of Intermediates **3a–d**

*tert*-Butyl-3-(benzyl(methyl)amino)pyrrolidine-1-carboxylate (**3a**). **3a** was prepared from *N*-Boc-pyrrolidin-3-one (**2a**) (1.5 g, 8.10 mmol), *N*,*N*-benzylmethylamine (1.1 g, 8.91 mmol), glacial AcOH (534.9 mg, 8.91 mmol) and Na(OAc)_3_BH (2.6 g, 12.15 mmol) in dry DCM (17 mL) according to general procedure A. Purification by flash column chromatography (SiO_2_, n-hexane:EtOAc 3:1) gave 1.8 g of a yellow oil (76% yield); ^1^H-NMR (300 MHz, CDCl_3_) δ 7.38–7.22 (m, 5H, overlap with CHCl_3_ signal), 3.79–3.46 (m, 4H), 3.36–3.15 (m, 2H), 3.11–2.94 (m, 1H), 2.16 (s, 3H), 2.14–2.05 (m, 1H), 1.98–1.79 (m, 1H), 1.48 (s, 9H); ^13^C-NMR (75 MHz, CDCl_3_) δ 154.6, 138.7, 138.6, 129.2, 129.0, 128.4, 127.2, 79.3, 64.0, 63.2, 60.5, 50.0, 49.6, 45.2, 44.8, 39.8, 30.1, 29.1, 28.6; GC-MS *method A*: t_r_ = 8.792 min, (*m/z*) 290 [M].

*tert*-Butyl-3-(benzyl(methyl)amino)piperidine-1-carboxylate (**3b**). **3b** was prepared from *N*-Boc-piperidin-3-one (**2b**) (2.5 g, 12.55 mmol), *N*,*N*-benzylmethylamine (2.0 g, 16.31 mmol), glacial acetic acid (904.1 mg, 15.06 mmol) and Na(OAc)_3_BH (4.3 g, 20.08 mmol) in dry DCM (30 mL) according to general procedure A. After stirring overnight reaction control indicated incomplete conversion, therefore a second portion of *N*,*N*-benzylmethylamine (494.1 mg, 4.08 mmol) and Na(OAc)_3_BH (1.1 g, 5.02 mmol) was added and stirring continued for 1 h. Purification by flash column chromatography (SiO_2_, petroleum ether:EtOAc 3:1) gave 2.6 g of a yellow oil (69% yield); ^1^H-NMR (300 MHz, CDCl_3_) δ 7.32–7.07 (m, 5H, overlap with CHCl_3_ signal), 4.35–3.78 (m, 2H), 3.59 (d, *J* = 13.5 Hz, 1H), 3.51 (d, *J* = 13.4 Hz, 1H), 2.74–2.49 (m, 2H), 2.46–2.31 (m, 1H), 2.15 (s, 3H), 1.96– 1.82 (m, 1H), 1.70–1.57 (m, 1H), 1.48–1.29 (m, 11H); ^13^C-NMR (75 MHz, CDCl_3_) δ 155.0, 139.7, 128.8, 128.3, 126.9, 79.4, 59.4, 58.4, 46.2 (br), 44.4 (br), 38.0, 28.5, 27.6 (br), 24.7 (br).

*tert*-Butyl-4-(benzyl(methyl)amino)azepane-1-carboxylate (**3c**). **3c** was prepared from *N*-Boc-hexahydro-1*H*-azepin-4-one (**2c**) (2.25 g, 10.55 mmol), *N*,*N*-benzylmethylamine (1.5 g, 12.66 mmol), glacial AcOH (696.9 mg, 11.61 mmol) and Na(OAc)_3_BH (3.4 g, 15.83 mmol) in dry DCM (25 mL) according to general procedure A. After stirring overnight reaction control indicated incomplete conversion, therefore a second portion of *N*,*N*-benzylmethylamine (639.2 mg, 5.27 mmol) and Na(OAc)_3_BH (1.1 g, 5.27 mmol) was added and stirring continued for 1 d. Purification by flash column chromatography (SiO_2_, n-hexane:EtOAc 3:1) gave 2.7 g of a yellow oil (81% yield); ^1^H-NMR (300 MHz, CDCl_3_) δ 7.36–7.20 (m, 5H, overlap with CHCl_3_ signal), 3.66–3.39 (m, 4H), 3.32–3.17 (m, 2H), 2.67–2.56 (m, 1H), 2.21–2.16 (m, 3H), 2.10–1.80 (m, 3H), 1.78–1.41 (m, 12H); ^13^C-NMR (75 MHz, CDCl_3_) δ 155.6, 140.0, 140.0, 128.7, 128.3, 126.9, 126.9, 79.2, 63.2, 63.1, 57.8, 57.7, 46.8, 46.4, 44.3, 44.0, 37.6, 30.3, 29.9, 29.8, 28.6, 25.9.

*tert*-Butyl-4-(benzyl(methyl)amino)piperidine-1-carboxylate (**3d**). **3d** was prepared from *N*-Boc-piperidin-4-one (**2d**) (2.75 g, 13.80 mmol), *N*,*N*-benzylmethylamine (1.8 g, 15.18 mmol), glacial AcOH (911.7 mg, 15.18 mmol) and Na(OAc)_3_BH (4.4 g, 20.70 mmol) in dry DCM (30 mL) according to general procedure A. The reaction mixture was stirred over molecular sieves instead of Na_2_SO_4_, which was separated by filtration before stopping the reaction with saturated NaHCO_3_. Purification by flash column chromatography (SiO_2_, petroleum ether:EtOAc:3.5N NH_3_ in MeOH 25:73:2) gave 3.6 g of a white solid (86% yield) ^1^H-NMR (200 MHz, CDCl_3_) δ 7.37–7.17 (m, 5H, overlap with CHCl_3_ signal), 4.28–4.04 (m, 2H), 3.59 (s, 2H), 2.80–2.50 (m, 3H), 2.21 (s, 3H), 1.90–1.73 (m, 2H), 1.62–1.43 (m, 11H); ^13^C-NMR (50 MHz, CDCl_3_) δ 154.9, 139.5, 128.9, 128.4, 127.1, 79.6, 60.9, 58.1, 43.6, 37.7, 28.6, 28.0; GC-MS *method A*: t_r_ = 9.663 min, (*m/z*) 304 [M].

##### Detailed Procedures for the Preparation of Intermediates **4a–d**

*tert*-Butyl-3-(methylamino)pyrrolidine-1-carboxylate (**4a**). **3a** (1.6 g, 5.51 mmol) was dissolved in HPLC grade MeOH (30 mL) and Pd/C 10% (*m/m*) (532.0 mg) was added. The suspension was stirred in a reactor charged with 5 bar of H_2_ pressure at rt for 4 h and then filtered over a pad of celite rinsing with fresh solvent. The filtrate was concentrated under reduced pressure to give 1.1 g of a green oil (96% crude yield), which was used in the next step without further purification; ^1^H-NMR (400 MHz, CDCl_3_) δ 3.48–3.19 (m, 3H), 3.17–2.93 (m, 2H), 2.33 (s, 3H), 2.00–1.88 (m, 1H), 1.67–1.54 (m, 1H), 1.38–1.33 (m, 9H), 1.30 (br s, 1H); ^13^C-NMR (101 MHz, CDCl_3_) δ 154.6, 79.0, 59.5, 58.7, 51.6, 51.1, 44.3, 44.0, 34.7, 31.7, 31.0, 28.5; GC-MS *method A*: t_r_ = 3.039 min, (*m/z*) 200 [M].

*tert*-Butyl-3-(methylamino)piperidine-1-carboxylate (**4b**). **3b** (2.2 g, 7.16 mmol) was dissolved in a solvent mixture of EtOAc (27 mL) and MeOH (18 mL). Pd/C 10% (*m/m*) (727.0 mg) was added and the mixture stirred in a reactor charged with 5 bar of H_2_ pressure at rt for 3 h. The mixture was filtered over a pad of celite rinsing with fresh solvent. The filtrate was concentrated under reduced pressure to give 1.5 g of a green oil (96% crude yield), which was used in the next step without further purification; ^1^H-NMR (400 MHz, CDCl_3_) δ 4.18–3.67 (m, 2H), 2.99–2.51 (m, 2H), 2.49–2.37 (m, 4H), 1.95–1.85 (m, 1H), 1.70–1.60 (m, 1H), 1.49–1.37 (m, 10H), 1.35–1.21 (m, 2H); ^13^C-NMR (101 MHz, CDCl_3_) δ 155.0, 79.5, 55.6, 48.8 (br), 44.3 (br), 33.9, 31.3, 28.6, 23.6 (br). GC-MS *method B*: t_r_ = 11.729 min, (*m/z*) 214 [M].

*tert*-Butyl-4-(methylamino)azepane-1-carboxylate (**4c**). **3c** (662.0 mg, 2.08 mmol) was dissolved in a solvent mixture of EtOAc (9 mL) and MeOH (6 mL). Pd/C 10% (*m/m*) (220.7 mg) was added and the mixture stirred in a reactor charged with 5 bar of H_2_ pressure at rt for 3 h. The mixture was filtered over a pad of celite rinsing with fresh solvent. The filtrate was concentrated under reduced pressure to give 463 mg of a green oil (98% crude yield), which was used in the next step without further purification; ^1^H-NMR (400 MHz, CDCl_3_) δ 3.54–3.36 (m, 2H), 3.36–3.09 (m, 2H), 2.51–2.41 (m, 1H), 2.36 (s, 3H), 1.97–1.87 (m, 1H), 1.87–1.72 (m, 2H), 1.59–1.31 (m, 13H); ^13^C-NMR (101 MHz, CDCl_3_) δ 155.6, 79.2, 60.2, 59.8, 46.6, 46.0, 43.3, 42.8, 34.8, 34.5, 34.2, 33.2, 32.5, 28.6, 24.8, 24.4. GC-MS *method B*: t_r_ = 13.406 min, (*m/z*) 228 [M].

*tert*-Butyl-4-(methylamino)piperidine-1-carboxylate (**4d**). **3d** (5.7 g, 18.72 mmol) was dissolved in MeOH (150 mL). Pd/C 10% (*m/m*) (600 mg) and Pd(OH)_2_/C 20% (*m/m*) (300 mg) were added and the mixture stirred in a reactor charged with 5 bar of H_2_ pressure at rt overnight. The mixture was filtered over a pad of celite rinsing with fresh solvent. The filtrate was concentrated under reduced pressure to give 3.9 g of a green oil (97% crude yield), which was used in the next step without further purification; 1H-NMR (200 MHz, CDCl_3_) δ 4.17–3.83 (m, 2H), 2.88–2.65 (m, 2H), 2.55–2.42 (m, 1H), 2.40 (s, 3H), 1.92–1.74 (m, 2H), 1.42 (s, 9H), 1.33–1.04 (m, 2H); ^13^C-NMR (50 MHz, CDCl_3_) δ 155.0, 79.5, 56.8, 42.6 (br), 33.5, 32.2, 28.5; GC-MS *method A*: t_r_ = 3.499 min, (*m/z*) 214 [M].

##### Detailed Procedures for the Preparation of 4,7-dichloro-9-tosyl-9*H*-pyrimido[4,5-*b*]indole (**10**)

Ethyl-2-(4-chloro-2-nitrophenyl)-2-cyanoacetate (**6**). A solution of ethyl-2-cyanoacetate (12.4 g, 109.38 mmol) in dry DMF (10 mL) was drop-added to a stirring, ice-cooled suspension of NaH (4.4 g of a 60% dispersion in mineral oil, 109.38 mmol) in dry DMF (20 mL). After complete addition, the dropping funnel was purged with additional dry DMF (5 mL) and ice-cooling was removed. After stirring at rt for 0.5 h, a solution of 1,4-dichloro-2-nitrobenzene (**5**) (10.0 g, 52.08 mmol) in dry DMF (10 mL) was drop-added. The stirring mixture was subsequently heated to 80 °C for 0.5 h, when reaction control via HPLC indicated complete conversion. The mixture was left to cool to rt and acidified with 10% HCl_(aq)_ (50 mL). EtOAc (100 mL) was added, phases were separated and the aqueous layer extracted with additional EtOAc (3 × 30 mL). Combined organic layers were washed with saturated NaCl solution (5 × 50 mL) and dried over Na_2_SO_4_. The mixture was concentrated under reduced pressure and the liquid residue treated with ice-cold water and stirred with ice-cooling. The resulting yellow precipitate was triturated with the ice-cold water, filtered washing with ice-cold water and dried over P_2_O_5_ in vacuo. 14.5 g of a yellow solid (> 100% crude yield) that may contain traces of excessive ethyl-2-cyanoacetate, but was used in the next step without further purification; ^1^H-NMR (200 MHz, DMSO-*d_6_*) δ 8.35 (d, *J* = 2.0 Hz, 1H), 8.02 (dd, *J* = 8.2, 2.1 Hz, 1H), 7.78 (d, *J* = 8.2 Hz, 1H), 6.27 (s, 1H), 4.22 (q, *J* = 7.1 Hz, 2H), 1.19 (t, *J* = 7.1 Hz, 3H); ^13^C-NMR (50 MHz, DMSO-*d_6_*) δ 163.7, 147.4, 135.2, 134.9, 134.6, 126.0, 124.5, 115.0, 63.1, 40.7, 13.8; ESI-MS: (*m/z*) 266.9 [M − H]^−^; HPLC: t_r_ = 6.655 min.

Ethyl-2-amino-6-chloro-1*H*-indole-3-carboxylate (**7**). **6** (7.0 g, 26.06 mmol) was dissolved in glacial AcOH (60 mL). The solution was stirred at 85 °C and Zinc dust (20.4 g, 312.72 mmol) was added in ten portions. The suspension was stirred at 85 °C for 75 min when reaction control via HPLC indicated complete consumption of the starting material. After cooling to rt, Zn dust was filtered off rinsing with AcOH (or EtOAc, alternatively) and the filtrate was concentrated under reduced pressure to leave a liquid residue. Careful addition of saturated NaHCO_3_ solution neutralized residual AcOH resulting in a precipitate which was filtered off, washed with water and dried over P_2_O_5_ in vacuo. 5.8 g of a red-brown solid (93% crude yield), which was used in the next step without further purification. A small batch was purified by flash column chromatography (SiO_2_, DCM/MeOH 97.5:2.5) for analytical purposes. ^1^H-NMR shows a mixture of products, which are assumed to be the title compound and the corresponding 1-hydroxyindole [27]; HPLC: t_r_ = 8.042 min.

7-Chloro-3,9-dihydro-4*H*-pyrimido[4,5-*b*]indol-4-one (**8**). **7** (5.7 g, 23.88 mmol) and NH_4_HCO_2_ (1.7 g, 27.46 mmol) were suspended in formamide (50 mL) and stirred at 160 °C for 28 h with reflux cooling when reaction control via HPLC indicated nearly full consumption of the starting material. After cooling to rt the mixture was poured into ice-cold water resulting in a precipitate which was filtered, washed thoroughly with ice-cold water and dried over P_2_O_5_ in vacuo. 4.5 g of a green-brown solid (86% crude yield), which was used in the next step without further purification; ^1^H-NMR (200 MHz, DMSO-*d_6_*) δ 12.32 (br s, 2H), 8.15 (s, 1H), 7.95 (d, *J* = 8.5 Hz, 1H), 7.48 (d, *J* = 1.4 Hz, 1H), 7.25 (dd, *J* = 8.5, 1.5 Hz, 1H); ^13^C-NMR (50 MHz, DMSO-*d_6_*) δ 158.0, 154.3, 148.0, 136.0, 128.4, 121.8, 121.4, 120.9, 111.4, 100.0; ESI-MS: (*m/z*) 217.9 [M − H]^−^; HPLC: t_r_ = 5.489 min.

4,7-Dichloro-9*H*-pyrimido[4,5-*b*]indole (**9**). **8** (4.5 g, 20.44 mmol) was suspended in chlorobenzene (30 mL) and DIPEA (4.0 g, 30.66 mmol) was added. The mixture was stirred at rt and under N_2_ atmosphere when POCl_3_ (4.4 g, 28.62 mmol) was added carefully dropwise. After stirring at rt for 1 h, the mixture was heated to 80 °C for additional 4.5 h with reflux cooling when HPLC indicated complete consumption of the starting material. The mixture was left to cool down and dropped carefully into stirring water (300 mL) at rt resulting in a brown precipitate. Saturated NaHCO_3_ solution was added carefully and the suspension left to stir overnight for neutralization. The precipitate was filtered, washed with water and dried over P_2_O_5_ in vacuo. 4.2 g of a brown solid (87% crude yield), which was purified by the following recrystallization procedure: 2 g of crude 4,7-dichloro-9*H*-pyrimido[4,5-*b*]indole were suspended in boiling toluene (650 mL) and stirred for 0.5 h. The hot suspension was filtered rinsing the brown filter cake with fresh hot toluene. The filtrate was concentrated under reduced pressure resulting in precipitation of the product. The suspension was cooled and subsequently the precipitate was filtered, washed with cold toluene and dried under reduced pressure giving 1.2 g of a yellow solid (59% recrystallization yield, 51% total yield); ^1^H-NMR (200 MHz, DMSO-*d_6_*) δ 12.85 (s, 1H), 8.76 (s, 1H), 8.14 (d, *J* = 8.5 Hz, 1H), 7.57 (d, *J* = 1.9 Hz, 1H), 7.37 (dd, *J* = 8.5, 1.9 Hz, 1H); ^13^C-NMR (50 MHz, DMSO-*d_6_*) δ 156.3, 154.2, 151.3, 139.1, 132.7, 123.6, 121.9, 116.5, 112.0, 110.7; ESI-MS: (*m/z*) 235.7 [M − H]^−^; HPLC: t_r_ = 8.590 min.

4,7-Dichloro-9-tosyl-9*H*-pyrimido[4,5-*b*]indole (**10**). NaH (151.2 mg of a 60% dispersion in mineral oil, 3.78 mmol) was added in three portions to a stirring suspension of 4,7-dichloro-9*H*-pyrimido[4,5-*b*]indole (**9**) (600.0 mg, 2.52 mmol) in dry THF (20 mL). p-Toluenesulfonyl chloride (576.6 mg, 3.02 mmol) was added after 20 min and stirring continued for another 0.5 h at rt and under N_2_ atmosphere when TLC indicated complete consumption of the starting material. The mixture was poured into ice-cold water and saturated NH_4_Cl solution (60 mL) was added. The precipitate was filtered, washed with cold water and dried over P_2_O_5_ in vacuo. 982 mg of a yellow solid (99% crude yield), which was directly used in the next step without further purification; ESI-MS: (*m/z*) 390.0 [M − H]^−^.

##### Detailed Procedures for the Preparation of Intermediates **11a**–**d**

*tert*-Butyl-3-((7-chloro-9-tosyl-9*H*-pyrimido[4,5-*b*]indol-4-yl)(methyl)amino)pyrrolidine-1-carboxyla-te (**11a**). **11a** was prepared from **10** (1.0 g, 2.55 mmol), **4a** (638.4 mg, 3.19 mmol) and DIPEA (988.8 mg, 7.65 mmol) in dry DMF (25 mL) according to general procedure B. 1.4 g of a beige solid (>100% crude yield), which was used in the next step without further purification. A small batch was purified by flash column chromatography (SiO_2_, DCM:MeOH 97.5:2.5) for analytical purposes; ^1^H-NMR (300 MHz, CDCl_3_) δ 8.64 (s, 1H), 8.54 (d, *J* = 1.8 Hz, 1H), 8.10 (d, *J* = 8.1 Hz, 2H), 7.63 (d, *J* = 8.5 Hz, 1H), 7.40 (dd, *J* = 8.5, 1.9 Hz, 1H), 7.27 (d, 2H, overlap with CHCl_3_ signal), 4.93–4.74 (m, 1H), 3.94–3.75 (m, 1H), 3.71–3.53 (m, 1H), 3.48–3.29 (m, 2H), 3.10 (s, 3H), 2.37 (s, 3H), 2.33–2.06 (m, 2H), 1.46 (s, 9H); ^13^C-NMR (50 MHz, CDCl_3_) δ 161.2, 157.2, 154.5, 154.3, 145.8, 136.2, 135.3, 132.9, 129.8, 128.1, 124.5, 123.3, 119.9, 114.7, 102.0, 79.6, 58.0, 57.4, 47.8, 44.7, 44.3, 35.2, 28.7, 28.6, 28.0, 21.7; ESI-MS: (*m/z*) 578.1 [M + Na]^+^, 554.3 [M − H]^−^; HPLC: t_r_ = 11.123 min.

*tert*-Butyl-3-((7-chloro-9-tosyl-9*H*-pyrimido[4,5-*b*]indol-4-yl)(methyl)amino)piperidine-1-carboxylate (**11b**). **11b** was prepared from **10** (430.0 mg, 1.10 mmol), **4b** (328.9 mg, 1.54 mmol) and DIPEA (425.1 mg, 3.29 mmol) in dry DMF (12 mL) according to general procedure B. 580 mg of a beige solid (93% crude yield), which was used in the next step without further purification. A small batch was purified by flash column chromatography (SiO_2_, DCM:MeOH 97.5:2.5) for analytical purposes; ^1^H-NMR (300 MHz, CDCl_3_) δ 8.60 (s, 1H), 8.52 (d, *J* = 1.9 Hz, 1H), 8.09 (d, *J* = 8.4 Hz, 2H), 7.60 (d, *J* = 8.1 Hz, 1H), 7.38 (dd, *J* = 8.5, 1.9 Hz, 1H), 7.25 (d, *J* = 8.1 Hz, 2H, overlap with CHCl_3_ signal), 4.49–3.96 (m, 3H), 3.10 (s, 3H), 3.08–2.98 (m, 1H), 2.76–2.61 (m, 1H), 2.35 (s, 3H), 2.00–1.71 (m, 3H), 1.63–1.50 (m, 1H), 1.39 (s, 9H); ESI-MS: (*m/z*) 569.9 [M + H]^+^, 591.9 [M + Na]^+^, 568.0 [M − H]^-^; HPLC: t_r_ = 11.456 min.

*tert*-Butyl-4-((7-chloro-9-tosyl-9*H*-pyrimido[4,5-*b*]indol-4-yl)(methyl)amino)azepane-1-carboxylate (**11c**). **11c** was prepared from **10** (465.0 mg, 1.19 mmol), **4c** (338.4 mg, 1.48 mmol) and DIPEA (459.7 mg, 3.56 mmol) in dry DMF (14 mL) according to general procedure B. 682 mg of a beige solid (98% crude yield), which was used in the next step without further purification. A small batch was purified by flash column chromatography (SiO_2_, DCM:MeOH 97.5:2.5) for analytical purposes; ^1^H-NMR (400 MHz, DMSO-*d_6_*) δ 8.49 (s, 1H), 8.36 (d, *J* = 1.9 Hz, 1H), 8.07–8.01 (m, 2H), 7.79–7.73 (m, 1H), 7.52 (dd, *J* = 8.6, 1.6 Hz, 1H), 7.40 (d, *J* = 8.2 Hz, 2H), 4.28–4.18 (m, 1H), 3.49–3.35 (m, 2H), 3.29–3.12 (m, 2H), 3.04 (s, 3H), 2.33 (s, 3H), 1.96–1.76 (m, 5H), 1.67–1.53 (m, 1H), 1.36–1.28 (m, 9H); ESI-MS: (*m/z*) 606.0 [M + Na]^+^, 582.1 [M − H]^−^; HPLC: t_r_ = 11.827 min.

*tert*-Butyl-4-((7-chloro-9-tosyl-9*H*-pyrimido[4,5-*b*]indol-4-yl)(methyl)amino)piperidine-1-carboxylate (**11d**). **11d** was prepared from **10** (200.0 mg, 0.51 mmol), **4d** (142.1 mg, 0.66 mmol) and DIPEA (197.7 mg, 1.53 mmol) in dry DMF (5.5 mL) according to general procedure B. Beige solid (>100% crude yield), which was used in the next step without further purification. A small batch was purified by flash column chromatography (SiO_2_, petroleum ether:EtOAc gradient elution from 9:1 to 4:6) for analytical purposes; ^1^H-NMR (200 MHz, CDCl_3_) δ 8.59 (s, 1H), 8.52 (d, *J* = 1.8 Hz, 1H), 8.09 (d, *J* = 8.3 Hz, 2H), 7.54 (d, *J* = 8.6 Hz, 1H), 7.37 (dd, *J* = 8.5, 1.9 Hz, 1H), 7.26 (d, *J* = 8.2 Hz, 2H, overlap with CHCl_3_ signal), 4.49–4.33 (m, 1H), 4.33–4.16 (m, 2H), 3.07 (s, 3H), 2.94–2.68 (m, 2H), 2.36 (s, 3H), 1.92–1.72 (m, 4H), 1.47 (s, 9H); ^13^C-NMR (50 MHz, CDCl_3_) δ 160.1, 157.4, 154.8, 154.1, 145.8, 136.2, 135.5, 132.7, 129.8, 128.2, 124.4, 123.1, 120.5, 114.8, 100.8, 80.0, 56.6, 43.4 (br), 33.4, 29.0, 28.6, 21.8. ESI-MS: (*m/z*) 592.0 [M + Na]^+^, 568.0 [M − H]^-^; HPLC: t_r_ = 11.988 min.

##### Detailed Procedures for the Preparation of Intermediates **12a**–**d**

*tert*-Butyl-3-((7-chloro-9*H*-pyrimido[4,5-*b*]indol-4-yl)(methyl)amino)pyrrolidine-1-carboxylate (**12a**). **12a** was prepared from **11a** (697.0 mg, 1.26 mmol) and K*t*BuO (986.1 mg, 8.79 mmol) in dry THF (40 mL) according to general procedure C in a reaction time of 1.5 h. Purification by flash column chromatography (SiO_2_, DCM:MeOH gradient elution from 97.5:2.5 to 93:7) gave 394 mg of a beige solid (78% yield); ^1^H-NMR (300 MHz, CDCl_3_) δ 11.68 (br s, 1H), 8.65–8.51 (m, 1H), 7.74 (d, *J* = 8.6 Hz, 1H), 7.53 (d, *J* = 1.8 Hz, 1H), 7.29 (dd, 1H, overlap with CHCl_3_ signal), 5.23–5.02 (m, 1H), 3.99–3.80 (m, 1H), 3.78–3.37 (m, 3H), 3.30 (s, 3H), 2.39–2.14 (m, 2H), 1.49 (s, 9H); ^13^C-NMR (50 MHz, DMSO-*d_6_*) δ 160.2, 157.4, 153.7, 153. 6, 137.5, 129.5, 124.0, 120.6, 118.3, 110.9, 98.1, 78.4, 56.8, 56.2, 47.0, 44.4, 44.1, 34.1, 28.2, 27.9, 27.0; ESI-MS: (*m/z*) 424.2 [M + Na]^+^, 400.2 [M − H]^−^; HPLC: t_r_ = 9.653 min.

*tert*-Butyl-3-((7-chloro-9*H*-pyrimido[4,5-*b*]indol-4-yl)(methyl)amino)piperidine-1-carboxylate (**12b**). **12b** was prepared from **11b** (580.0 mg, 1.02 mmol) and K*t*BuO (799.1 mg, 7.12 mmol) in dry THF (32 mL) according to general procedure C in a reaction time of 1 h. Purification by flash column chromatography (SiO_2_, DCM:MeOH gradient elution from 97.5:2.5 to 93:7) gave 320 mg of a beige solid (76% yield); ^1^H-NMR (200 MHz, CDCl_3_) δ 12.25 (br s, 1H), 8.57 (s, 1H), 7.70 (d, *J* = 8.6 Hz, 1H), 7.50 (d, *J* = 1.8 Hz, 1H), 7.24 (dd, 1H, overlap with CHCl_3_ signal), 4.59–3.95 (m, 3H), 3.26 (s, 3H), 3.17–2.96 (m, 1H), 2.84–2.56 (m, 1H), 2.19–1.55 (m, 4H), 1.43 (s, 9H); ^13^C-NMR (50 MHz, CDCl_3_) δ 160.2, 157.2, 155.0, 152.5, 137.5, 131.1, 123.7, 121.5, 118.8, 111.6, 98.7, 80.0, 55.2, 46.8, 44.1, 33.4, 28.5, 28.2, 25.0; ESI-MS: (*m/z*) 438.0 [M + Na]^+^, 413.9 [M − H]^−^; HPLC: t_r_ = 9.001 min.

*tert*-Butyl-4-((7-chloro-9*H*-pyrimido[4,5-*b*]indol-4-yl)(methyl)amino)azepane-1-carboxylate (**12c**). **12c** was prepared from **11c** (682.0 mg, 1.17 mmol) and K*t*BuO (917.0 mg, 8.17 mmol) in dry THF (40 mL) following general procedure C in a reaction time of 0.75 h. Purification by flash column chromatography (SiO_2_, DCM:MeOH gradient elution from 97.5:2.5 to 93:7) gave 392 mg of a beige solid (78% yield); ^1^H-NMR (400 MHz, CDCl_3_) δ 12.43 (br s, 1H), 8.55 (s, 1H), 7.69–7.62 (m, 1H), 7.50–7.45 (m, 1H), 7.25–7.20 (m, 1H), 4.64–4.52 (m, 1H), 3.88–3.78 (m, 0.5H), 3.72–3.63 (m, 0.5H), 3.57–3.38 (m, 2H), 3.28–3.15 (m, 4H), 2.11–1.67 (m, 6H), 1.47 (s, 9H); ^13^C-NMR (101 MHz, CDCl_3_) δ 159.9, 157.3, 155.7, 152.7, 137.5, 130.9, 123.5, 121.3, 119.0, 111.6, 98.4, 79.6, 58.9, 58.4, 46.9, 46.0, 43.6, 43.5, 33.1, 32.3, 31.82, 31.79, 31.5, 28.7, 25.43, 25.36; ESI-MS: (*m/z*) 430.0 [M + H]^+^, 452.0 [M + Na]^+^, 428.0 [M − H]^−^; HPLC: t_r_ = 9.734 min.

*tert*-Butyl-4-((7-chloro-9*H*-pyrimido[4,5-*b*]indol-4-yl)(methyl)amino)piperidine-1-carboxylate (**12d**). **12d** was prepared from **11d** (290.8 mg, 0.51 mmol) and K*t*BuO (400.7 mg, 3.57 mmol) in HPLC grade THF (32 mL) according to general procedure C in a reaction time of 2 h. Purification by flash column chromatography (SiO_2_, DCM:MeOH gradient elution from 97.5:2.5 to 93:7) gave 138 mg of an off-white solid (65% yield); ^1^H-NMR (400 MHz, CDCl_3_) δ 12.09 (s, 1H), 8.56 (s, 1H), 7.67 (d, *J* = 8.6 Hz, 1H), 7.48 (d, *J* = 1.9 Hz, 1H), 7.24 (dd, *J* = 8.6, 1.9 Hz, 1H), 4.70–4.58 (m, 1H), 4.46–4.11 (m, 2H), 3.23 (s, 3H), 2.99–2.75 (m, 2H), 1.96–1.80 (m, 4H), 1.50 (s, 9H); ^13^C-NMR (50 MHz, CDCl_3_) δ 160.0, 157.2, 154.9, 152.5, 137.5, 130.9, 123.4, 121.2, 118.9, 111.6, 98.3, 79.9, 55.9, 43.6, 33.2, 29.1, 28.6; ESI-MS: (*m/z*) 416.1 [M + H]^+^, 438.1 [M + Na]^+^, 414.1 [M − H]^−^; HPLC: t_r_ = 10.236 min.

##### Detailed Procedures for the Preparation of Intermediates **13a–d**

7-Chloro-*N*-methyl-*N*-(pyrrolidin-3-yl)-9*H*-pyrimido[4,5-*b*]indol-4-amine (**13a**). **13a** was prepared from **12a** (340.0 mg, 0.85 mmol) and TFA (1.8 mL) in dry DCM (9 mL) according to general procedure D. 220 mg of a beige solid (86% yield), which was used in the next step without further purification; ^1^H-NMR (300 MHz, DMSO-*d_6_*) δ 8.40 (s, 1H), 7.83 (d, *J* = 8.6 Hz, 1H), 7.48 (d, *J* = 2.0 Hz, 1H), 7.27 (dd, *J* = 8.6, 2.0 Hz, 1H), 4.97–4.85 (m, 1H), 3.17 (s, 3H), 3.14–3.07 (m, 1H), 3.00–2.75 (m, 3H), 2.12–1.98 (m, 1H), 1.94–1.80 (m, 1H). Both N-H not detected due to hydrogen bonding; ^13^C-NMR (75 MHz, DMSO-*d_6_*) δ 160.1, 157.4, 153.7, 137.4, 129.2, 123.8, 120.4, 118.6, 110.8, 97.6, 58.8, 49.2, 46.4, 33.4, 29.0; ESI-MS: (*m/z*) 301.9 [M + H]^+^, 299.9 [M − H]*^−^*, HPLC: t_r_ = 3.556 min.

7-Chloro-*N*-methyl-*N*-(piperidin-3-yl)-9*H*-pyrimido[4,5-*b*]indol-4-amine (**13b**). **13b** was prepared from **12b** (314.0 mg, 0.76 mmol) and TFA (1.7 mL) in dry DCM (8.5 mL) according to general procedure D in a reaction time of 1 h. 236 mg of a beige solid (99% yield), which was used in the next step without further purification; ^1^H-NMR (300 MHz, MeOD) δ 8.34 (s, 1H), 7.75 (d, *J* = 8.6 Hz, 1H), 7.49 (d, *J* = 1.9 Hz, 1H), 7.26 (dd, *J* = 8.6, 2.0 Hz, 1H), 4.48 (tt, *J* = 11.2, 4.0 Hz, 1H), 3.25 (s, 3H), 3.17–3.08 (m, 1H), 3.05–2.96 (m, 1H), 2.96–2.86 (m, 1H), 2.63–2.49 (m, 1H), 2.16–1.85 (m, 3H), 1.78–1.60 (m, 1H); ^13^C-NMR (50 MHz, DMSO-*d_6_*) δ 159.6, 157.5, 153.7, 137.4, 129.1, 123.8, 120.4, 118.7, 110.8, 97.1, 55.7, 48.6, 45.5, 32.7, 28.2, 26.4; ESI-MS: (*m/z*) 316.0 [M + H]^+^, 338.0 [M + Na]^+^, 313.9 [M − H]^-^; HPLC: t_r_ = 3.815 min.

*N*-(Azepan-4-yl)-7-chloro-*N*-methyl-9*H*-pyrimido[4,5-*b*]indol-4-amine (**13c**). **13c** was prepared from **12c** (125.0 mg, 0.29 mmol) and TFA (1 mL) in dry DCM (5 mL) according to general procedure D in a reaction time of 1.5 h. Purification by flash column chromatography (SiO_2_, DCM:2N NH_3_ in MeOH 9:1) gave 71 mg of a beige solid (74% yield); ^1^H-NMR (400 MHz, CDCl_3_) δ 8.53 (s, 1H), 7.66 (d, *J* = 8.6 Hz, 1H), 7.44 (d, *J* = 1.6 Hz, 1H), 7.20 (dd, *J* = 8.6, 1.8 Hz, 1H), 4.75–4.66 (m, 1H), 3.23 (s, 3H), 3.12–2.99 (m, 2H), 2.96–2.87 (m, 2H), 2.13–1.96 (m, 4H), 1.95–1.85 (m, 1H), 1.78–1.66 (m, 1H). Both N-H not detected due to hydrogen bonding; ^13^C-NMR (101 MHz, CDCl_3_) δ 160.1, 157.8, 153.1, 137.5, 130.7, 123.5, 121.1, 119.2, 111.5, 98.5, 58.6, 48.9, 46.1, 35.0, 32.7, 31.6, 27.6; ESI-MS: (*m/z*) 329.9 [M + H]^+^, 328.0 [M − H]^-^; HPLC: t_r_ = 3.401 min.

7-Chloro-*N*-methyl-*N*-(piperidin-4-yl)-9*H*-pyrimido[4,5-*b*]indol-4-amine (**13d**). **13d** was prepared from **12d** (105.0 mg, 0.25 mmol) and TFA (0.6 mL) in dry DCM (3 mL) according to general procedure D in a reaction time of 1 h. 73 mg of a beige solid (92% yield) which was used in the next step without further purification; ^1^H-NMR (400 MHz, MeOD) δ 8.34 (s, 1H), 7.73 (d, *J* = 8.6 Hz, 1H), 7.50 (d, *J* = 1.9 Hz, 1H), 7.26 (dd, *J* = 8.6, 2.0 Hz, 1H), 4.55–4.46 (m, 1H), 3.23 (s, 3H), 3.21–3.13 (m, 2H), 2.76–2.66 (m, 2H), 2.02–1.90 (m, 2H), 1.90–1.83 (m, 2H); ESI-MS: (*m/z*) 316.0 [M + H]^+^, 314.0 [M − H]^-^; HPLC: t_r_ = 3.485 min.

##### Detailed Procedures for the Preparation of Compounds **14a–o**

3-(3-((7-Chloro-9*H*-pyrimido[4,5-*b*]indol-4-yl)(methyl)amino)piperidin-1-yl)-3-oxopropanenitrile (**14a**). Cyanoacetic acid (23.1 mg, 0.27 mmol) and PyBOP (141.4 mg, 0.27 mmol) were dissolved in dry DCM (5 mL) and stirred at rt and under N_2_ atmosphere for 20 min. A suspension of **13b** (71.5 mg, 0.23 mmol) and DIPEA (87.8 mg, 0.68 mmol) in dry DCM (5 mL) was added and the mixture stirred at rt and under N_2_ atmosphere for 1.5 h. The mixture was then diluted with DCM, washed with saturated NaHCO_3_ solution (4 × 15 mL), dried over Na_2_SO_4_ and concentrated under reduced pressure. Purification of the residue by flash column chromatography (SiO_2_, 1. DCM:MeOH gradient elution from 95:5 to 92.5:7.5, 2. DCM:EtOH gradient elution from 96.5:3.5 to 93:7) gave 51 mg of a white solid (59% yield); NMR shows a 7:3 mixture of amide bond rotamers, ^1^H (400 MHz, CDCl_3_) δ 12.59 (br s, 1H), 8.51–8.30 (m, 1H), 7.67–7.48 (m, 1H), 7.41–7.30 (m, 1H), 7.21–7.09 (m, 1H), 4.90–4.78 (m, 0.3H), 4.70–4.58 (m, 0.7H), 4.45–4.23 (m, 1H), 4.22–4.11 (m, 0.7H), 4.04–3.79 (m, 1.4H), 3.78–3.69 (m, 0.3H), 3.66–3.54 (m, 0.6H), 3.30–3.12 (m, 4H), 3.05–2.94 (m, 0.3H), 2.70–2.56 (m, 0.7H), 2.26–1.88 (m, 3H), 1.86–1.64 (m, 1H); ^13^C-NMR (101 MHz, CDCl_3_) δ 161.2, 160.6, 159.8, 157.2, 156.7, 152.5, 152.0, 137.5, 137.4, 131.24, 131.17, 123.6, 123.5, 121.5, 121.4, 118.41, 118.35, 114.7, 114.1, 111.7, 111.6, 98.5, 77.4, 55.5, 54.6, 48.2, 46.9, 45.4, 43.5, 34.9, 34.0, 28.7, 27.6, 25.40, 25.37, 25.2, 24.8; ESI-MS: (*m/z*) 383.1 [M + H]^+^, 405.1[M + Na]^+^, 380.9 [M − H]^-^; HPLC: t_r_ = 6.202 min (100.0% purity).

3-(3-((7-Chloro-9*H*-pyrimido[4,5-*b*]indol-4-yl)(methyl)amino)piperidin-1-yl)propanenitrile (**14b**). **13b** (125.0 mg, 0.40 mmol) and acrylonitrile (46.2 mg, 0.87 mmol) were stirred in dry MeOH (35 mL) at rt and under N_2_ atmosphere overnight. Volatiles were removed under reduced pressure. Purification of the residue by flash column chromatography (SiO_2_, DCM:MeOH gradient elution from 95.5:4.5 to 93.5:6.5) gave 92 mg of a white solid (63% yield); ^1^H-NMR (400 MHz, DMSO-*d_6_*) δ 12.22 (s, 1H), 8.40 (s, 1H), 7.78 (d, *J* = 8.6 Hz, 1H), 7.48 (s, 1H), 7.34 (d, *J* = 8.4 Hz, 1H), 4.56–4.33 (m, 1H), 3.14 (s, 3H), 3.11–3.03 (m, 1H), 2.92–2.81 (m, 1H), 2.78–2.68 (m, 2H), 2.68–2.58 (m, 2H), 2.44–2.32 (m, 1H), 2.02–1.88 (m, 1H), 1.84–1.63 (m, 3H), 1.59–1.41 (m, 1H); ^13^C-NMR (101 MHz, DMSO-*d_6_*) δ 159.4, 157.4, 153.7, 137.3, 129.1, 123.8, 120.6, 120.0, 118.5, 110.8, 97.0, 55.7, 54.7, 53.0, 52.2, 32.5, 27.2, 24.3, 15.0. ESI-MS: (*m/z*) 369.1 [M + H]^+^, 391.0 [M + Na]^+^, 366.9 [M − H]^-^; HPLC: t_r_ = 3.695 min (100.0% purity).

3-(3-((7-Chloro-9*H*-pyrimido[4,5-*b*]indol-4-yl)(methyl)amino)piperidin-1-yl)propanamide (**14c**). **13b** (65.0 mg, 0.21 mmol) and acrylamide (16.1 mg, 0.23 mmol) were stirred in dry MeOH (11 mL) at rt and under N_2_ atmosphere overnight. Additional acrylamide (16.1 mg, 0.23 mmol) was added and stirring at rt continued for 5 days. Volatiles were removed under reduced pressure. Purification of the residue by flash column chromatography (SiO_2_, DCM:2N NH_3_ in MeOH gradient elution from 92:8 to 9:1) gave 65 mg of a white solid (82% yield); ^1^H-NMR (400 MHz, DMSO-*d_6_*) δ 12.21 (s, 1H), 8.39 (s, 1H), 7.76 (d, *J* = 8.7 Hz, 1H), 7.48 (d, *J* = 2.0 Hz, 1H), 7.34 (br s, 1H), 7.30 (dd, *J* = 8.6, 2.0 Hz, 1H), 6.78 (br s, 1H), 4.50–4.34 (m, 1H), 3.15 (s, 3H), 3.05–2.96 (m, 1H), 2.87–2.77 (m, 1H), 2.65–2.52 (m, 2H), 2.35–2.18 (m, 3H), 1.96–1.85 (m, 1H), 1.84–1.65 (m, 3H), 1.58–1.42 (m, 1H); ^13^C-NMR (101 MHz, DMSO-*d_6_*) δ 173.2, 159.4, 157.4, 153.7, 137.3, 129.1, 123.8, 120.5, 118.5, 110.8, 97.0, 55.9, 54.8, 54.2, 52.7, 33.0, 32.6, 27.4, 24.4; ESI-MS: (*m/z*) 387.4 [M + H]^+^, 409.4 [M + Na]^+^, 385.3 [M − H]^−^; HPLC: t_r_ = 3.872 min (100.0% purity).

Methyl-3-(3-((7-chloro-9*H*-pyrimido[4,5-*b*]indol-4-yl)(methyl)amino)piperidin-1-yl)propanoate (**14d**). **13b** (65.0 mg, 0.21 mmol) and methyl acrylate (19.5 mg, 0.23 mmol) were stirred in dry MeOH (11 mL) at rt and under N_2_ atmosphere for 3 h. Additional methyl acrylate (4.4 mg, 0.05 mmol) was added and stirring at rt continued for 1 h. Volatiles were removed under reduced pressure. Purification of the residue by flash column chromatography (SiO_2_, DCM:MeOH gradient elution from 96:4 to 92:8) gave 74 mg of a beige solid (89% yield); ^1^H-NMR (400 MHz, DMSO-*d_6_*) δ 12.21 (s, 1H), 8.39 (s, 1H), 7.75 (d, *J* = 8.7 Hz, 1H), 7.47 (d, *J* = 2.0 Hz, 1H), 7.28 (dd, *J* = 8.6, 2.0 Hz, 1H), 4.45–4.34 (m, 1H), 3.55 (s, 3H), 3.14 (s, 3H), 3.00–2.92 (m, 1H), 2.84–2.75 (m, 1H), 2.62 (t, *J* = 6.9 Hz, 2H), 2.53–2.46 (m, 2H, overlap with DMSO-*d_5_* signal), 2.36–2.26 (m, 1H), 1.95–1.85 (m, 1H), 1.83–1.65 (m, 3H), 1.55–1.41 (m, 1H); ^13^C-NMR (101 MHz, DMSO-*d_6_*) δ 172.4, 159.4, 157.4, 153.7, 137.3, 129.1, 123.8, 120.4, 118.5, 110.8, 97.0, 55.9, 54.8, 53.3, 52.5, 51.1, 32.6, 31.6, 27.3, 24.4; ESI-MS: (*m/z*) 402.5 [M + H]^+^, 424.6 [M + Na]^+^, 400.3 [M − H]^-^; HPLC: t_r_ = 4.363 min (100.0% purity).

7-Chloro-*N*-methyl-*N*-(1-propylpiperidin-3-yl)-9*H*-pyrimido[4,5-*b*]indol-4-amine (**14e**). **14e** was prepared from **13b** (65.0 mg, 0.21 mmol), propionaldehyde (17.9 mg, 0.31 mmol), glacial AcOH (24.7 mg, 0.41 mmol) and Na(OAc)_3_BH (87.2 mg, 0.41 mmol) in dry DCM (10 mL) according to general procedure E in a reaction time of 2 h. Purification by flash column chromatography (SiO_2_, 1. DCM:2N NH_3_ in MeOH gradient elution from 95:5 to 92:8, 2. DCM:2N NH_3_ in MeOH gradient elution from 94:6 to 91.5:8.5) gave 46 mg of a white solid (62% yield); ^1^H-NMR (400 MHz, DMSO-*d_6_*) δ 2.21 (s, 1H), 8.39 (s, 1H), 7.78 (d, *J* = 8.7 Hz, 1H), 7.47 (d, *J* = 2.0 Hz, 1H), 7.23 (dd, *J* = 8.6, 2.0 Hz, 1H), 4.48–4.37 (m, 1H), 3.14 (s, 3H), 3.00–2.91 (m, 1H), 2.84–2.76 (m, 1H), 2.33–2.17 (m, 3H), 1.87–1.66 (m, 4H), 1.58–1.39 (m, 3H), 0.84 (t, *J* = 7.3 Hz, 3H); ^13^C-NMR (101 MHz, DMSO-*d_6_*) δ 159.4, 157.4, 153.7, 137.3, 129.1, 123.8, 120.3, 118.6, 110.8, 97.0, 60.0, 56.3, 54.9, 52.9, 32.6, 27.6, 24.5, 19.6, 11.7; ESI-MS: (*m/z*) 358.2 [M + H]^+^, 380.1 [M + Na]^+^, 356.2 [M − H]^-^; HPLC: t_r_ = 4.200 min (99.3% purity).

7-Chloro-*N*-(1-isopropylpiperidin-3-yl)-*N*-methyl-9*H*-pyrimido[4,5-*b*]indol-4-amine (**14f**). **14f** was prepared from **13b** (62.0 mg, 0.20 mmol), acetone (228.1 mg, 3.93 mmol), glacial AcOH (23.6 mg, 0.39 mmol) and Na(OAc)_3_BH (83.2 mg, 0.39 mmol) in dry DCM (10 mL) according to general procedure E in a reaction time of 5 h. A second portion of acetone (228.1 mg, 3.93 mmol) was added after 3 h. Purification by flash column chromatography (SiO_2_, DCM:2N NH_3_ in MeOH 95:5 to 92.5:7.5) gave 30 mg of a beige solid (43% yield); ^1^H-NMR (400 MHz, DMSO-*d_6_*) δ 12.20 (s, 1H), 8.39 (s, 1H), 7.79 (d, *J* = 8.7 Hz, 1H), 7.47 (d, *J* = 2.0 Hz, 1H), 7.24 (dd, *J* = 8.6, 2.0 Hz, 1H), 4.46–4.35 (m, 1H), 3.16 (s, 3H), 2.96–2.86 (m, 1H), 2.79–2.66 (m, 2H), 2.48–2.41 (m, 1H), 2.16–2.03 (m, 1H), 1.86–1.67 (m, 3H), 1.55–1.41 (m, 1H), 1.05–0.92 (m, 6H); ^13^C-NMR (50 MHz, DMSO-*d_6_*) δ 159.4, 157.4, 153.8, 137.3, 129.1, 123.9, 120.3, 118.6, 110.8, 97.0, 55.5, 54.0, 51.3, 48.1, 32.7, 28.0, 24.8, 18.1, 17.7; ESI-MS: (*m/z*) 357.8 [M + H]^+^, 355.8 [M − H]^-^; HPLC: t_r_ = 4.291 min (99.0% purity).

7-Chloro-*N*-(1-cyclopentylpiperidin-3-yl)-*N*-methyl-9*H*-pyrimido[4,5-*b*]indol-4-amine (**14g**). **14g** was prepared from **13b** (60.0 mg, 0.19 mmol), cyclopentanone (63.9 mg, 0.76 mmol), glacial AcOH (22.8 mg, 0.38 mmol) and Na(OAc)_3_BH (80.5 mg, 0.38 mmol) in dry DCM (10 mL) according to general procedure E in a reaction time of 3 h. Purification by flash column chromatography (SiO_2_, DCM:2N NH_3_ MeOH gradient elution from 96:4 to 93:7) gave 54 mg of a white solid (74% yield); ^1^H-NMR (400 MHz, DMSO-*d_6_*) δ 12.21 (s, 1H), 8.38 (s, 1H), 7.79 (d, *J* = 8.7 Hz, 1H), 7.47 (d, *J* = 1.9 Hz, 1H), 7.24 (dd, *J* = 8.6, 2.0 Hz, 1H), 4.47–4.35 (m, 1H), 3.14 (s, 3H), 3.09–3.01 (m, 1H), 2.94–2.84 (m, 1H), 2.58–2.52 (m, 1H, overlap with DMSO-*d_5_*), 2.30–2.17 (m, 1H), 1.93–1.67 (m, 6H), 1.64–1.39 (m, 5H), 1.38–1.25 (m, 2H); ^13^C-NMR (101 MHz, DMSO-*d_6_*) δ 159.4, 157.4, 153.7, 137.3, 129.1, 123.8, 120.2, 118.6, 110.8, 96.9, 66.7, 55.0, 54.8, 51.5, 32.6, 29.8, 29.6, 27.6, 24.5, 23.6; ESI-MS: (*m/z*) 383.8 [M + H]^+^, 381.8 [M − H]^-^; HPLC: t_r_ = 4.839 min (100.0% purity).

7-Chloro-*N*-(1-(furan-2-ylmethyl)piperidin-3-yl)-*N*-methyl-9*H*-pyrimido[4,5-*b*]indol-4-amine (**14h**). **14h** was prepared from **13b** (80.0 mg, 0.25 mmol), furan-2-carbaldehyde (30.4 mg, 0.32 mmol), glacial AcOH (30.4 mg, 0.51 mmol) and Na(OAc)_3_BH (80.5 mg, 0.38 mmol) in dry DCM (10 mL) according to general procedure E in a reaction time of 6.5 h. Purification by flash column chromatography (SiO_2_, 1.DCM:MeOH gradient elution from 96:4 to 92.5:7.5, 2.DCM:MeOH gradient elution from 96:4 to 92:8) gave 55 mg of an off-white solid (55% yield); ^1^H-NMR (300 MHz, CDCl_3_) δ 11.76 (br s, 1H), 8.54 (s, 1H), 7.63 (d, *J* = 8.6 Hz, 1H), 7.49–7.37 (m, 2H), 7.21 (dd, *J* = 8.6, 1.8 Hz, 1H), 6.39–6.31 (m, 1H), 6.30–6.22 (m, 1H), 4.69–4.50 (m, 1H), 3.71 (d, *J* = 14.2 Hz, 1H), 3.64 (d, *J* = 14.2 Hz, 1H), 3.28–3.14 (m, 4H), 3.03–2.92 (m, 1H), 2.50–2.37 (m, 1H), 2.14–2.02 (m, 1H), 2.00–1.90 (m, 1H), 1.88–1.71 (m, 3H); ^13^C-NMR (50 MHz, CDCl_3_) δ 160.3, 157.6, 153.1, 151.4, 142.5, 137.3, 130.8, 123.7, 121.3, 119.0, 111.4, 110.4, 109.4, 98.7, 56.1, 55.7, 54.9, 53.2, 33.2, 28.0, 24.7.; ESI-MS: (*m/z*) 396.1 [M + H]^+^, 418.1 [M + Na]^+^, 393.9 [M − H]^-^; HPLC: t_r_ = 4.781 min (99.7% purity).

7-Chloro-*N*-methyl-*N*-(1-(pyridin-4-ylmethyl)piperidin-3-yl)-9*H*-pyrimido[4,5-*b*]indol-4-amine (**14i**). **14i** was prepared from **13b** (75.0 mg, 0.24 mmol), pyridin-4-carbaldehyde (38.2 mg, 0.36 mmol), glacial AcOH (28.5 mg, 0.48 mmol) and Na(OAc)_3_BH (100.7 mg, 0.48 mmol) in dry DCM (10 mL) according to general procedure E in a reaction time of 2.5 h. Purification by flash column chromatography (SiO_2_, DCM:MeOH gradient elution from 95:5 to 90.5:9.5) gave 71 mg of a white solid (73% yield); ^1^H-NMR (400 MHz, DMSO-*d_6_*) δ 12.22 (s, 1H), 8.49 (d, *J* = 5.7 Hz, 2H), 8.37 (s, 1H), 7.69 (d, *J* = 8.6 Hz, 1H), 7.48 (d, *J* = 1.8 Hz, 1H), 7.32 (d, *J* = 5.6 Hz, 2H), 7.22 (dd, *J* = 8.6, 1.8 Hz, 1H), 4.54–4.41 (m, 1H), 3.59–3.48 (m, 2H), 3.13 (s, 3H), 2.98–2.88 (m, 1H), 2.80–2.70 (m, 1H), 2.37–2.27 (m, 1H), 2.00–1.89 (m, 1H), 1.86–1.65 (m, 3H), 1.62–1.48 (m, 1H); ^13^C-NMR (50 MHz, DMSO-*d_6_*) δ 159.4, 157.5, 153.7, 149.5, 147.5, 137.4, 129.2, 123.8, 123.7, 120.3, 118.5, 110.9, 97.0, 60.8, 55.9, 54.8, 52.8, 32.8, 27.2, 24.4; ESI-MS: (*m/z*) 407.4 [M + H]^+^, 405.2 [M − H]^−^; HPLC: t_r_ = 6.121 min (99.5% purity).

3-((3-((7-Chloro-9*H*-pyrimido[4,5-*b*]indol-4-yl)(methyl)amino)piperidin-1-yl)methyl)benzonitrile (**14j**). **14j** was prepared from **13b** (25.0 mg, 0.08 mmol), 3-formylbenzonitrile (31.1 mg, 0.24 mmol), glacial AcOH (9.5 mg, 0.16 mmol) and Na(OAc)_3_BH (33.6 mg, 0.16 mmol) in dry DCM (4 mL) according to general procedure E in a reaction time of 4 h. Purification by flash column chromatography (SiO_2_, DCM:MeOH gradient elution from 96:4 to 93:7) gave 20 mg of a white solid (59% yield); ^1^H-NMR (400 MHz, DMSO-*d_6_*) δ 12.20 (s, 1H), 8.37 (s, 1H), 7.77 (br s, 1H), 7.75–7.64 (m, 3H), 7.54 (t, *J* = 7.7 Hz, 1H), 7.47 (d, *J* = 2.0 Hz, 1H), 7.21 (dd, *J* = 8.6, 2.0 Hz, 1H), 4.54–4.41 (m, 1H), 3.61 (d, *J* = 13.7 Hz, 1H), 3.54 (d, *J* = 13.7 Hz, 1H), 3.14 (s, 3H), 3.01–2.91 (m, 1H), 2.82–2.72 (m, 1H), 2.37–2.26 (m, 1H), 2.02–1.90 (m, 1H), 1.84–1.67 (m, 3H), 1.61–1.48 (m, 1H); ^13^C-NMR (50 MHz, DMSO-*d_6_*) δ 159.4, 157.4, 153.7, 140.2, 137.4, 133.7, 132.1, 130.9, 129.4, 129.2, 123.8, 120.3, 118.9, 118.5, 111.2, 110.9, 97.0, 61.0, 55.6, 54.8, 52.8, 32.8, 27.2, 24.4; ESI-MS: (*m/z*) 431.2 [M + H]^+^, 453.2 [M + Na]^+^, 429.2 [M − H]^-^; HPLC: t_r_ = 4.879 min (100.0% purity).

7-Chloro-*N*-methyl-*N*-(1-(3,3,3-trifluoropropyl)piperidin-3-yl)-9*H*-pyrimido[4,5-*b*]indol-4-amine (**14k**). **14k** was prepared from **13b** (55.0 mg, 0.17 mmol), trifluoropropanal (97.6 mg, 0.87 mmol), glacial AcOH (20.9 mg, 0.35 mmol) and Na(OAc)_3_BH (73.8 mg, 0.35 mmol) in dry DCM (10 mL) according to general procedure E in a reaction time of 3 h. Purification by flash column chromatography (SiO_2_, DCM:MeOH gradient elution from 97.5:2.5 to 92.5:7.5) gave 38 mg of a white solid (53% yield); ^1^H-NMR (400 MHz, DMSO-*d_6_*) δ 12.20 (s, 1H), 8.39 (s, 1H), 7.76 (d, *J* = 8.7 Hz, 1H), 7.48 (d, *J* = 2.0 Hz, 1H), 7.26 (dd, *J* = 8.6, 2.0 Hz, 1H), 4.49–4.37 (m, 1H), 3.15 (s, 3H), 3.03–2.96 (m, 1H), 2.88–2.79 (m, 1H), 2.61–2.53 (m, 2H), 2.51–2.41 (m, 2H, overlapping with DMSO-*d_5_*), 2.37–2.26 (m, 1H), 1.96–1.87 (m, 1H), 1.86–1.67 (m, 3H), 1.59–1.45 (m, 1H); ^13^C-NMR (101 MHz, DMSO-*d_6_*) δ 159.4, 157.4, 153.7, 137.3, 129.1, 127.2 (q, *J* = 276.9 Hz), 123.7, 120.3, 118.5, 110.8, 97.0, 55.8, 54.7, 52.4, 50.4 (q), 32.6, 30.4 (q, *J* = 26.3 Hz), 27.2, 24.3; ESI-MS: (*m/z*) 412.3 [M + H]^+^, 434.4 [M + Na]^+^, 410.3 [M − H]^-^; HPLC: t_r_ = 7.463 min (97.2% purity).

2-(3-((7-Chloro-9*H*-pyrimido[4,5-*b*]indol-4-yl)(methyl)amino)piperidin-1-yl)acetonitrile (**14l**). **13b** (40.0 mg, 0.13 mmol) was dissolved in dry DMF (2 mL). 2-Bromoacetonitrile (16.7 mg, 0.14 mmol) and Et_3_N (38.5 mg, 0.38 mmol) were added and the mixture stirred at rt for 1.5 h. Saturated NaHCO_3_ solution (5 mL) was added and the mixture extracted with EtOAc (6 × 2 mL). Combined organic layers were diluted with additional EtOAc, washed with saturated NaHCO_3_ solution (3 × 20 mL), dried over Na_2_SO_4_ and concentrated under reduced pressure. Purification of the residue by flash column chromatography (SiO_2_, DCM:MeOH 96:4) gave 41 mg of a beige solid (91% yield); ^1^H-NMR (400 MHz, DMSO-*d_6_*) δ 12.22 (s, 1H), 8.41 (s, 1H), 7.76 (d, *J* = 8.6 Hz, 1H), 7.48 (d, *J* = 1.9 Hz, 1H), 7.27 (dd, *J* = 8.6, 2.0 Hz, 1H), 4.52–4.41 (m, 1H), 3.75 (s, 2H), 3.18 (s, 3H), 2.95–2.87 (m, 1H), 2.81–2.72 (m, 1H), 2.48–2.42 (m, 1H), 2.20–2.09 (m, 1H), 1.95–1.72 (m, 3H), 1.66–1.53 (m, 1H); ^13^C-NMR (101 MHz, DMSO-*d_6_*) δ 159.5, 157.4, 153.7, 137.4, 129.2, 123.8, 120.4, 118.4, 115.8, 110.8, 97.2, 54.4, 54.1, 51.3, 45.3, 32.8, 26.6, 24.1; ESI-MS: (*m/z*) 355.0 [M + H]^+^, 376.9 [M + Na]^+^, 352.9 [M − H]^-^; HPLC: t_r_ = 8.358 min (99.4% purity).

7-Chloro-*N*-(1-(cyclopropylmethyl)piperidin-3-yl)-*N*-methyl-9*H*-pyrimido[4,5-*b*]indol-4-amine (**14m**). (Bromomethyl)cyclopropane (34.4 mg, 0.25 mmol) and Et_3_N (33.6 mg, 0.33 mmol) were added to a suspension of **13b** (70.0 mg, 0.22 mmol) in HPLC grade acetonitrile (10 mL). The mixture was stirred at 60 °C for 2 days and then concentrated under reduced pressure. Purification of the residue by flash column chromatography (SiO_2_, DCM:2N NH_3_ in MeOH gradient elution from 98:2 to 91.5:8.5) gave 64 mg of a white solid (78% yield); ^1^H-NMR (400 MHz, DMSO-*d_6_*) δ 12.22 (s, 1H), 8.39 (s, 1H), 7.80 (d, *J* = 8.7 Hz, 1H), 7.47 (d, *J* = 2.0 Hz, 1H), 7.25 (dd, *J* = 8.6, 2.0 Hz, 1H), 4.49–4.38 (m, 1H), 3.14 (s, 3H), 3.12–3.06 (m, 1H), 2.94–2.87 (m, 1H), 2.30–2.12 (m, 3H), 1.92–1.65 (m, 4H), 1.60–1.45 (m, 1H), 0.91–0.78 (m, 1H), 0.51–0.36 (m, 2H), 0.13–0.00 (m, 2H); ^13^C-NMR (101 MHz, DMSO-*d_6_*) δ 159.4, 157.4, 153.7, 137.3, 129.1, 123.8, 120.3, 118.6, 110.8, 97.0, 62.9, 56.1, 54.9, 52.8, 32.5, 27.6, 24.4, 8.3, 3.8, 3.6; ESI-MS: (*m/z*) 370.1 [M + H]^+^, 392.1 [M + Na]^+^, 368.2 [M − H]^-^; HPLC: t_r_ = 4.318 min (100.0% purity).

7-Chloro-*N*-(1-(3-(dimethylamino)propyl)piperidin-3-yl)-*N*-methyl-9*H*-pyrimido[4,5-*b*]indol-4-amine (**14n**). **13b** (100.0 mg, 0.32 mmol) and 3-chloro-*N*,*N*-dimethylpropan-1-amine hydrochloride (65.1 mg, 0.41 mmol) were suspended in HPLC grade acetonitrile (15 mL). Et_3_N (96.1 mg, 0.95 mmol) was added. The mixture was stirred at 90 °C for 2–3 days and then concentrated under reduced pressure. Purification of the residue by flash column chromatography (SiO_2_, DCM:2N NH_3_ in MeOH 9:1) gave 50 mg of an off-white solid (39% yield); ^1^H-NMR (300 MHz, CDCl_3_) δ 12.07 (br s, 1H), 8.54 (s, 1H), 7.69 (d, *J* = 8.6 Hz, 1H), 7.44 (d, *J* = 1.0 Hz, 1H), 7.21 (dd, *J* = 8.6, 1.2 Hz, 1H), 4.66–4.51 (m, 1H), 3.23 (s, 3H), 3.18–3.10 (m, 1H), 2.99–2.90 (m, 1H), 2.53–2.20 (m, 11H), 2.02–1.66 (m, 7H); ^13^C-NMR (101 MHz, CDCl_3_) δ 160.3, 157.8, 153.2, 137.5, 130.8, 123.7, 121.2, 119.2, 111.4, 98.6, 58.0, 57.0, 56.8, 55.6, 53.8, 45.3, 33.1, 28.5, 25.0, 24.9; ESI-MS: (*m/z*) 401.4 [M + H]^+^, 399.4 [M − H]^-^; HPLC: t_r_ = 4.318 min.

3-(3-((7-Chloro-9-methyl-9*H*-pyrimido[4,5-*b*]indol-4-yl)(methyl)amino)piperidin-1-yl)propanenitrile (**14o**). NaH (8.1 mg of a 60% dispersion in mineral oil, 0.20 mmol) was added to a stirring suspension of **14b** (50.0 mg, 0.14 mmol) in dry THF (10 mL). The mixture was stirred at rt and under N_2_ atmosphere for 0.5 h. Methyl iodide (0.5 mL of a freshly prepared 0.4M solution in THF, 0.20 mmol) was drop-added and the mixture stirred for 20 h at rt and under N_2_ atmosphere. Dimethylamine (0.075 mL of a 2M solution in THF, 0.15 mmol) was added to quench excessive methyl iodide and stirring continued for 1 h. Saturated NH_4_Cl solution (15 mL) was added to stop the reaction and the mixture then basified with saturated NaHCO_3_ solution. EtOAc was added and phases were separated. The organic layer was washed with saturated NaHCO_3_ solution (2 × 20 mL), dried over Na_2_SO_4_ and concentrated under reduced pressure. Purification of the residue by flash column chromatography (SiO_2_, DCM:MeOH 96.7:3.3 to 93.5:6.5) gave 31 mg of a light beige solid (60% yield); ^1^H-NMR (400 MHz, DMSO-*d_6_*) δ 8.46 (s, 1H), 7.83–7.74 (m, 2H), 7.38 (dd, *J* = 8.6, 1.3 Hz, 1H), 4.49–4.39 (m, 1H), 3.83 (s, 3H), 3.14 (s, 3H), 3.11–3.03 (m, 1H), 2.90–2.82 (m, 1H), 2.76–2.59 (m, 4H), 2.43–2.34 (m, 1H), 2.00–1.89 (m, 1H), 1.81–1.66 (m, 3H), 1.55–1.42 (m, 1H); ^13^C-NMR (101 MHz, DMSO-*d_6_*) δ 159.3, 156.8, 153.5, 138.5, 129.5, 123.7, 120.8, 120.0, 118.0, 109.7, 96.7, 55.6, 54.9, 53.0, 52.2, 32.6, 27.9, 27.2, 24.3, 15.0; ESI-MS: (*m/z*) 383.0 [M + H]^+^, 405.0 [M + Na]^+^; HPLC: t_r_ = 4.740 min (100.0% purity).

##### Detailed Procedures for the Preparation of Compounds **15–17**

3-(3-((7-Chloro-9*H*-pyrimido[4,5-*b*]indol-4-yl)(methyl)amino)pyrrolidin-1-yl)propanenitrile (**15**). **13a** (60.0 mg, 0.20 mmol) and acrylonitrile (23.2 mg, 0.44 mmol) were stirred in dry MeOH (17 mL) at rt overnight. Volatiles were removed under reduced pressure. Purification of the residue by flash column chromatography (SiO_2_, DCM:MeOH 96:4 to 93:7) gave 51 mg of an off-white solid (72% yield); ^1^H-NMR (400 MHz, DMSO-*d_6_*) δ 12.22 (s, 1H), 8.41 (s, 1H), 7.84 (d, *J* = 8.6 Hz, 1H), 7.48 (d, *J* = 1.9 Hz, 1H), 7.29 (dd, *J* = 8.6, 1.9 Hz, 1H), 5.11–5.01 (m, 1H), 3.24 (s, 3H), 2.98–2.86 (m, 2H), 2.78–2.57 (m, 5H), 2.43–2.33 (m, 1H), 2.27–2.16 (m, 1H), 2.05–1.94 (m, 1H); ^13^C-NMR (101 MHz, DMSO-*d_6_*) δ 160.0, 157.3, 153.7, 137.4, 129.2, 123.8, 120.4, 119.8, 118.5, 110.8, 97.6, 56.8, 56.2, 52.9, 50.3, 33.3, 27.8, 16.5; ESI-MS: (*m/z*) 355.0 [M + H]^+^, 377.0 [M + Na]^+^, 353.0 [M − H]^-^; HPLC: t_r_ = 3.414 min (100.0% purity).

3-(4-((7-Chloro-9*H*-pyrimido[4,5-*b*]indol-4-yl)(methyl)amino)azepan-1-yl)propanenitrile (**16**). **13c** (65.0 mg, 0.20 mmol) and acrylonitrile (23.0 mg, 0.43 mmol) were stirred in HPLC grade MeOH (20 mL) at rt and under N_2_ overnight. Additional acrylonitrile was added repeatedly, but conversion seized at ~ 80% as calculated by HPLC. Volatiles were removed under reduced pressure. Purification of the residue by flash column chromatography (SiO_2_, DCM:MeOH 95:5) gave 50 mg of a beige solid (66% yield); ^1^H-NMR (400 MHz, CDCl_3_) δ 12.59 (br s, 1H), 8.53 (s, 1H), 7.65 (d, *J* = 8.5 Hz, 1H), 7.44 (s, 1H), 7.21 (d, *J* = 8.5 Hz, 1H), 4.74–4.62 (m, 1H), 3.22 (s, 3H), 2.91 (t, *J* = 6.8 Hz, 2H), 2.87–2.71 (m, 4H), 2.52 (t, *J* = 6.6 Hz, 2H), 2.13–1.87 (m, 5H), 1.80–1.67 (m, 1H); ^13^C-NMR (101 MHz, CDCl_3_) δ 160.0, 157.7, 153.0, 137.4, 130.8, 123.5, 121.1, 119.1, 119.0, 111.5, 98.4, 58.4, 54.8, 54.0, 51.7, 32.9, 32.5, 30.9, 25.7, 16.8; ESI-MS: (*m/z*) 405.1 [M + Na]^+^, 381.1 [M − H]^-^; HPLC: t_r_ = 3.102 min (98.4% purity).

3-(4-((7-Chloro-9*H*-pyrimido[4,5-*b*]indol-4-yl)(methyl)amino)piperidin-1-yl)propanenitrile (**17**). **13d** (34.0 mg, 0.11 mmol) and acrylonitrile (8.6 mg, 0.16 mmol) were stirred in dry MeOH (10 mL) at rt and under N_2_ atmosphere overnight. Volatiles were removed under reduced pressure. Purification of the residue by flash column chromatography (SiO_2_, DCM:MeOH 97:3 to 95:5) gave 31 mg of a pale yellow solid (78% yield); ^1^H-NMR (200 MHz, DMSO-*d_6_*) δ 12.19 (s, 1H), 8.39 (s, 1H), 7.74 (d, *J* = 8.7 Hz, 1H), 7.48 (d, *J* = 1.9 Hz, 1H), 7.26 (dd, *J* = 8.6, 2.0 Hz, 1H), 4.41–4.19 (m, 1H), 3.14 (s, 3H), 3.07–2.91 (m, 2H), 2.73–2.53 (m, 4H), 2.19–1.65 (m, 6H); ^13^C-NMR (50 MHz, DMSO-*d_6_*) δ 159.6, 157.5, 153.8, 137.4, 129.2, 123.7, 120.4, 120.1, 118.6, 110.9, 97.2, 55.5, 52.7, 52.2, 32.5, 28.3, 15.2; ESI-MS: (*m/z*) 369.0 [M + H]^-^; 391.0 [M + Na]^+^, 367.0 [M − H]^-^; HPLC: t_r_ = 3.474 min (100.0% purity).

##### Detailed Procedures for the Preparation of Intermediates **19–23** and Compound **24**

*tert*-Butyl-1*H*-pyrrolo[2,3-*c*]pyridine-1-carboxylate (**19**). A solution of 1*H*-pyrrolo[2,3-*c*]pyridine (**18**) (500.0 mg, 4.23 mmol) in dry THF (5 mL) was stirred under N_2_ atmosphere and with ice-cooling. Boc anhydride (1108.4 mg, 5.08 mmol) was added dropwise. The mixture was left to warm to rt and stirred overnight under N_2_ atmosphere. After diluting with EtOAc the mixture was washed with saturated NaHCO_3_ solution (2 × 20 mL) and saturated NaCl solution (20 mL), dried over Na_2_SO_4_ and concentrated under reduced pressure. Purification of the residue by flash column chromatography (SiO_2_, DCM:MeOH 97.5:2.5) gave 846 mg of a yellow oil (92% yield); ^1^H-NMR (300 MHz, DMSO-*d_6_*) δ 9.25 (s, 1H), 8.34 (d, *J* = 5.3 Hz, 1H), 7.86 (d, *J* = 3.6 Hz, 1H), 7.63 (dd, *J* = 5.3, 1.1 Hz, 1H), 6.77 (dd, *J* = 3.6, 0.6 Hz, 1H), 1.64 (s, 9H); ^13^C-NMR (75 MHz, DMSO-*d_6_*) δ 148.4, 141.7, 136.5, 135.5, 131.5, 129.5, 115.7, 106.5, 84.8, 27.6; HPLC: t_r_ = 2.259 min.

*tert*-Butyl-octahydro-1*H*-pyrrolo[2,3-*c*]pyridine-1-carboxylate (**20**). **19** (746.0 mg, 3.42 mmol) was dissolved in glacial AcOH (50 mL) and PtO_2_ (150.0 mg, 20% (*m/m*)) was added. The mixture was stirred in a reactor charged with 5 bar of H_2_ pressure at rt for 35 h, then diluted with EtOAc and filtered over a pad of celite rinsing with EtOAc. The filtrate was concentrated under reduced pressure, redissolved in DCM and washed with saturated NaHCO_3_ solution (2 × 20 mL) adding saturated NaCl solution to improve phase separation. Combined aqueous layers were re-extracted with DCM (25 mL). Combined organic layers were dried over Na_2_SO_4_ and concentrated under reduced pressure to give 680 mg of a yellow oil (88% crude yield), which was used in the next step without further purification; GC-MS *method A*: t_r_ = 4.974 min, (*m/z*) 226 [M].

*tert*-Butyl-6-(2-cyanoethyl)octahydro-1*H*-pyrrolo[2,3-*c*]pyridine-1-carboxylate (**21**). **20** (340.0 mg, 1.50 mmol) was dissolved in HPLC grade MeOH (50 mL) and acrylonitrile (175.4 mg, 3.31 mmol) was added. The mixture was stirred at rt and under N_2_ atmosphere overnight. Volatiles were removed under reduced pressure. Purification of the residue by flash column chromatography (SiO_2_, petroleum ether:EtOAc 35:65) gave 350 mg of a yellow oil (83% yield); ^1^H-NMR (300 MHz, CDCl_3_) δ 3.91–3.73 (m, 1H), 3.43–3.18 (m, 2H), 2.99–2.86 (m, 1H), 2.65 (t, *J* = 7.2 Hz, 2H), 2.60–2.48 (m, 1H), 2.44 (t, *J* = 6.8 Hz, 2H), 2.33–1.54 (m, 7H), 1.40 (s, 9H); ^13^C-NMR (75 MHz, CDCl_3_) δ 154.5, 154.3, 118.9, 118.8, 79.3, 79.1, 55.0, 53.8, 53.4, 53.1, 49.2, 48.6, 45.4, 44.9, 35.1, 34.5, 28.5, 26.7, 25.8, 25.7, 25.5, 15.8; GC-MS *method A*: t_r_ = 8.701 min, (*m/z*) 279 [M].

3-(Octahydro-6*H*-pyrrolo[2,3-c]pyridin-6-yl)propanenitrile (**22**). **21** (300.0 mg, 1.07 mmol) was dissolved in dry DCM (6 mL) under Ar atmosphere. 4N HCl in dioxane (2.7 mL) was added to the stirring solution resulting in a waxy precipitate. The reaction progress was monitored by a ninhydrin stained TLC. After full consumption of **21**, demineralized water was added to dissolve the waxy precipitate. The pH of the aqueous layer was adjusted to 14 with 50% NaOH_(aq)_. Phases were separated and the aqueous layer was extracted with DCM (15 × 15 mL). Combined organic layers were dried over Na_2_SO_4_ and concentrated under reduced pressure. 160 mg of an orange oil (83% yield), which was used in the next step without further purification. A partial hydrolysis of the nitrile group to an amide group was observed in the NMR spectra of the crude product. A small batch was purified by flash column chromatography for analytical purposes (SiO_2_, DCM:2N NH_3_ in MeOH 9:1); ^1^H-NMR (400 MHz, CDCl_3_) δ 3.82–3.52 (m, 1H), 3.22–3.13 (m, 1H), 3.12–3.05 (m, 1H), 3.04–2.95 (m, 1H), 2.94–2.88 (m, 1H), 2.72–2.60 (m, 3H), 2.49 (t, *J* = 6.9 Hz, 2H), 2.45–2.38 (m, 1H), 2.16–2.07 (m, 1H), 2.01–1.93 (m, 1H), 1.91–1.82 (m, 1H), 1.64–1.54 (m, 2H), 1.49–1.40 (m, 1H); ^13^C-NMR (75 MHz, CDCl_3_) δ 118.9, 57.8, 53.7, 53.3, 51.9, 44.2, 35.8, 31.2, 27.1, 16.0; GC-MS method B: t_r_ = 14.130 min, (*m/z*) 179 [M].

3-(1-(7-Chloro-9-tosyl-9*H*-pyrimido[4,5-*b*]indol-4-yl)octahydro-6*H*-pyrrolo[2,3-c]pyridin-6-yl)propa-nenitrile (**23**). **10** (200.0 mg, 0.51 mmol), **22** (109.7 mg, 0.61 mmol) and DIPEA (210.9 mg, 1.63 mmol) were mixed with dry DMF (6 mL). The mixture was stirred at 80 °C for 1 h and was then left to cool to rt. Saturated NaCl solution (15 mL) was added and the mixture extracted with EtOAc (3 × 25 mL). Combined organic layers were washed with saturated NaCl solution (3 × 20 mL), dried over Na_2_SO_4_ and concentrated under reduced pressure. Purification of the residue by flash column chromatography (SiO_2_, petroleum ether:(EtOAc + MeOH 95 + 5) 4:6) gave 175 mg of a beige solid (64% yield); ^1^H-NMR (300 MHz, CDCl_3_) δ 8.56–8.48 (m, 2H), 8.10 (d, *J* = 8.4 Hz, 2H), 7.77 (br s, 1H), 7.36 (dd, *J* = 8.5, 1.2 Hz, 1H), 7.27 (d, 2H, overlapping with CHCl_3_ signal), 4.65–4.48 (m, 1H), 4.24–4.06 (m, 1H), 3.53–3.41 (m, 1H), 3.17–3.01 (m, 1H), 2.82–2.52 (m, 4H), 2.49–2.27 (m, 7H), 1.98–1.69 (m, 4H); ^13^C-NMR (75 MHz, CDCl_3_) δ 158.0, 156.9, 154.2, 145.7, 136.0, 135.6, 132.2, 129.8, 128.2, 124.2, 123.5, 120.7, 118.8, 114.5, 100.3, 57.5, 53.5, 53.4, 50.8, 34.6, 29.8, 26.8, 21.8, 15.8. Signal overlap assumed at 50.8; ESI-MS: (*m/z*) 534.9 [M + H]^+^, 556.8 [M + Na]^+^, 532.9 [M − H]^-^; HPLC: t_r_ = 7.197 min.

3-(3a*RS*,7a*SR*)-(1-(7-Chloro-9*H*-pyrimido[4,5-*b*]indol-4-yl)octahydro-6*H*-pyrrolo[2,3-c]pyridin-6-yl)-propanenitrile (**24**). **23** (150.0 mg, 0.28 mmol) was dissolved in dry THF (10 mL) and K*t*BuO (220.2 mg, 1.96 mmol) was added. The mixture was stirred at rt and under N_2_ atmosphere for 75 min. Saturated NaCl solution (25 mL) was added and the mixture extracted with EtOAc (3 × 25 mL). Combined organic layers were dried over Na_2_SO_4_ and concentrated under reduced pressure. Purification of the residue by flash column chromatography (SiO_2_, DCM:MeOH 95:5) gave 59 mg of a light beige solid (55% yield); ^1^H-NMR (300 MHz, pyridine-*d_5_*) δ 13.68 (br s, 1H), 8.84 (s, 1H), 8.23 (d, *J* = 8.8 Hz, 1H), 7.81 (d, *J* = 2.0 Hz, 1H), 7.52 (dd, *J* = 8.7, 2.0 Hz, 1H), 4.95–4.86 (m, 1H), 4.42–4.22 (m, 1H), 4.03–3.86 (m, 1H), 3.18–3.02 (m, 1H), 2.74–2.53 (m, 5H), 2.50–2.25 (m, 3H), 1.98–1.62 (m, 4H); ^13^C-NMR (101 MHz, DMSO-*d_6_*) δ 156.9, 156.6, 153.8, 137.3, 128.7, 123.8, 120.3, 119.9, 118.5, 110.7, 96.0, 56.6, 53.2, 52.9, 49.1, 48.4, 34.1, 26.7, 25.5, 14.9; ESI-MS: (*m/z*) 381.2 [M + H]^+^, 403.2 [M + Na]^+^, 379.2 [M − H]^−^; HPLC: t_r_ = 4.362 min (99.9% purity). Crystals suitable for X-ray determination were obtained by slow evaporation of a solution of **24** in methanol and chloroform at 298 K under atmospheric pressure. CCDC 1,917,242 contains the supplementary crystallographic data. These data can be obtained free of charge via http://www.ccdc.cam.ac.uk/conts/retrieving.html.

## Figures and Tables

**Table 1:** Structures, biological activity and calculated pK_a_ values of piperidine derivatives **14a**–**o**.

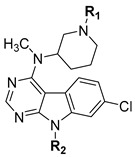				
Cpd.	R_1_	R_2_	IC_50_ (µM) Mean ± SEM GSK-3β ^a^	pK_a_^c^
**14a**	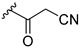	-H	1.862 ± 0.113	-
**14b**	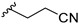	-H	0.764 ± 0.203 ^b^	5.5
**14c**	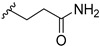	-H	>10	6.8
**14d**	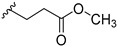	-H	>10	6.8
**14e**	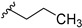	-H	>10	7.5
**14f**	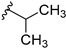	-H	>10	8.3
**14g**	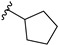	-H	>10	8.0
**14h**	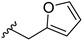	-H	>10	6.9
**14i**	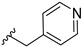	-H	>10	6.2
**14j**	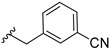	-H	>10	6.3
**14k**	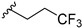	-H	>10	5.7
**14l**	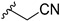	-H	>10	2.5
**14m**	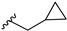	-H	>10	7.8
**14n**	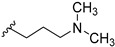	-H	3.557 ± 0.664	7.2
**14o**	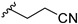	-CH_3_	>10	n.d. ^d^

^a^ IC_50_ values were determined in an ADP Glo kinase assay [21,22], *n* = 2; ^b^ *n* = 5; ^c^ pK_a_ value for the piperidine nitrogen calculated with Jaguar (Schrödinger LLC) (for details see Materials and Methods); ^d^ not determined.

**Table 2:** Structures, biological activity and AlogP-derived lipophilic ligand efficiencies (cLLE) of compounds **14b**, **15**–**17** and **24**.

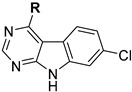					
Cpd.	R	IC_50_ (µM) Mean ± SEM GSK-3β ^a^	pIC_50_	AlogP ^d^	cLLE ^e^
**14b**	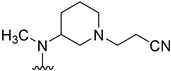	0.764 ± 0.203 ^b^	6.12	3.25	2.87
**15**	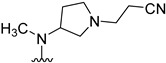	7.017 ± 1.077	5.15	2.67	2.48
**16**	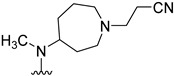	1.535 ± 0.284	5.81	3.31	2.50
**17**	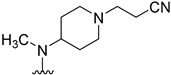	13.335 ± 4.393	4.88	2.73	2.15
**24**	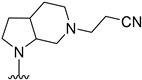	0.130 ± 0.008 ^c^	6.89	3.15	3.74

^a^ IC_50_ values were determined in an ADP Glo assay [21,22], *n* = 2; ^b^ *n* = 5; ^c^ *n* = 3; ^d^ calculated with Canvas (Schrödinger LLC) [23]; ^e^ cLLE = pIC_50_ − AlogP.

## Supplementary Materials

The following are available online at: Comparison of JAK3 and GSK-3β; MD Simulation of the (3a*R*, 7a*S*)-enantiomer of compound **24**; ATP binding competition of **14b**; JAK3 inhibition by compounds **14b** and **24**; metabolism in HLM of compounds **14b** and **24**; structure determination of compound **24**. The MD-movies, full-length raw-trajectories and the conformations used for pK_a_ calculations are freely available at https://doi.org/10.5281/zenodo.3248885.
