# Supplementary material for: Design, Synthesis and Biological Evaluation of 7-Chloro-9H-pyrimido[4,5-b]indole-based Glycogen Synthase Kinase-3β Inhibitors
Source: Molecules. 2019 Jun 25;24(12):2331. doi: 10.3390/molecules24122331 (PMC6630214; doi:10.3390/molecules24122331)
Supplement: Supplementary file 1 [file molecules-24-02331-s001.pdf]

Supplementary Materials

Design, Synthesis and Biological Evaluation of 7-Chloro-9*H*-pyrimido[4,5-*b*]indole-based Glycogen Synthase Kinase-3β inhibitors.

Stanislav Andreev [1], Tatu Pantsar [2,3], Francesco Ansideri [1], Mark Kudolo [1], Michael Forster [1], Dieter Schollmeyer [4], Stefan A. Laufer [1] and Pierre Koch [1,5,*]

[1] Institute of Pharmaceutical Sciences, Department of Medicinal and Pharmaceutical Chemistry, Eberhard Karls University Tübingen, Auf der Morgenstelle 8, 72076 Tübingen, Germany
[2] Department of Internal Medicine VIII, University Hospital Tübingen, Otfried-Müller-Str. 14, 72076 Tübingen, Germany
[3] School of Pharmacy, University of Eastern Finland, P.O. Box 1627, 70211 Kuopio, Finland
[4] Department of Organic Chemistry, Johannes Gutenberg University Mainz, Duesbergweg 10-14, 55099 Mainz, Germany
[5] Department of Pharmaceutical / Medicinal Chemistry II, Institute of Pharmacy, University of Regensburg, Universitätsstraße 31, 93053 Regensburg, Germany

* Correspondence: pierre.koch@uni-tuebingen.de; Tel.: +49-7071-29-74579.

Table of Contents

Comparison of JAK3 and GSK-3β S2

MD Simulation of the (3a*R*, 7a*S*)-Enantiomer of Compound **24** S3

ATP Binding Competition of Compound **14b** S4

JAK3 Inhibition by Compounds **14b** and **24** S5

Metabolism in Human Liver Microsomes (HLM) of Compounds **14b** and **24** S6

Structure Determination of Compound **24** S8

Comparison of JAK3 and GSK-3β

(a)

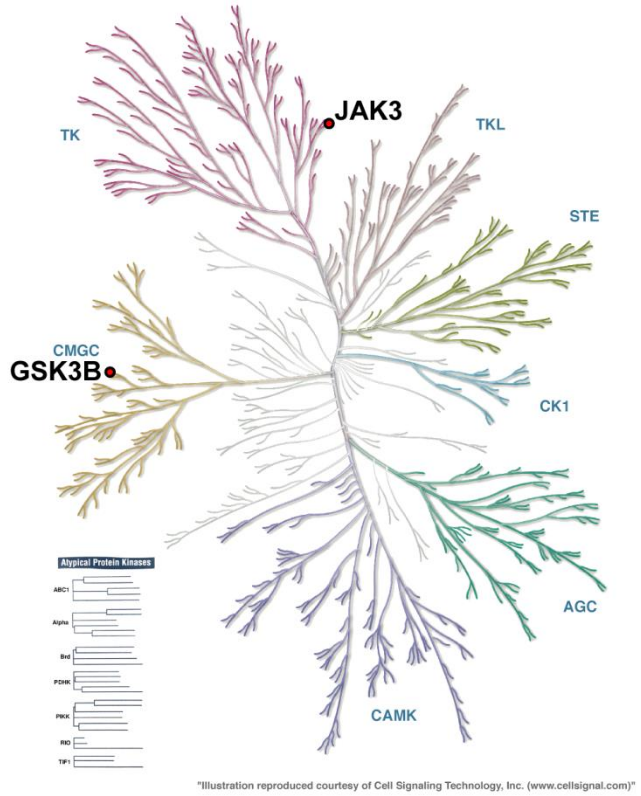

(b)

Kinase domain alignment of JAK3 and GSK3B

```
                Glycine-rich loop
JAK3  LKYISQLGKGNFGSVELCRYDPLGDNTGALVAVKQLQHSGPDQQRDFQREIQILKALHSD 881
GSK3B YTDTKVIGNGSFGVVYQAKL----CDSGELVAIKKVLQD----KRFKNRELQIMRKLDHC 107
       .  . :*:*.** *  .:      ::* ***:*:: :.    :*  :**:**:: *.

                                Hinge
JAK3  FIVKYRGVSYGPGRQ----SLRLVMEYLPSGCLRDFLQ--RHRARLDASRLLLYSSQICK 935
GSK3B NIVRLRYFFYSSGEKKDEVYLNLVLDYVPETVYRVARHYSRAKQTLPVIYVKLYMYQLFR 167
       **: * . *. *.:     *.**::*:*.   *   :  * :  * .  : **  *: :

                                       DFG
JAK3  GMEYLGSRRCVHRDLAARNILVESE-AHVKIADFGLAKLLPLDKDYYVVREPGQSPIFWY 994
GSK3B SLAYIHSFGICHRDIKPQNLLLDPDTAVLKLCDFGSAKQLVRGEPNVSY----ICSRYYR 223
      .: *: *    ***:  :*:*:: : * :*:.*** ** *  .:          .  ::

JAK3  APES-LSDNIFSRQSDVWSFGVVLYELFTYCDK-----SCSPSAEFLRMMGCERDVPALC 1048
GSK3B APELIFGATDYTSSIDVWSAGCVLAELLLGQPIFPGDSGVDQLVEIIKVLGTPTRE---- 279
      ***  :. . :: . **** * ** **:          . .  .*:::::*

JAK3  RLLELLEEGQRLPAPPACPAEVHELMKLCWAPSPQ--DRPSFSALGPQLD-----MLWSG 1101
GSK3B QI--------REMNPNYTEFKFPQIKAHPWTKVFRPRTPPEAIALCSRLLEYTPTARLTP 331
      ::        *   *     :. ::    *:   :    *.  **  :*         :

JAK3  SRGCETHAFT 1111
GSK3B LEACAHSFF- 340
       ..*    *

* = identical residue
: = residues with strongly similar properties
. = residues with weakly similar properties
```

Figure S1. (**a**) The locations of JAK3 and GSK-3β in the phylogenetic tree of the human kinome. (**b**) Sequence alignment of the kinase domains of JAK3 (residues: 822-1111; Uniprot: P52333) and GSK-3β (residues 56-340; Uniprot: P49841).

MD Simulation of the (3a*R*, 7a*S*)-Enantiomer of Compound 24

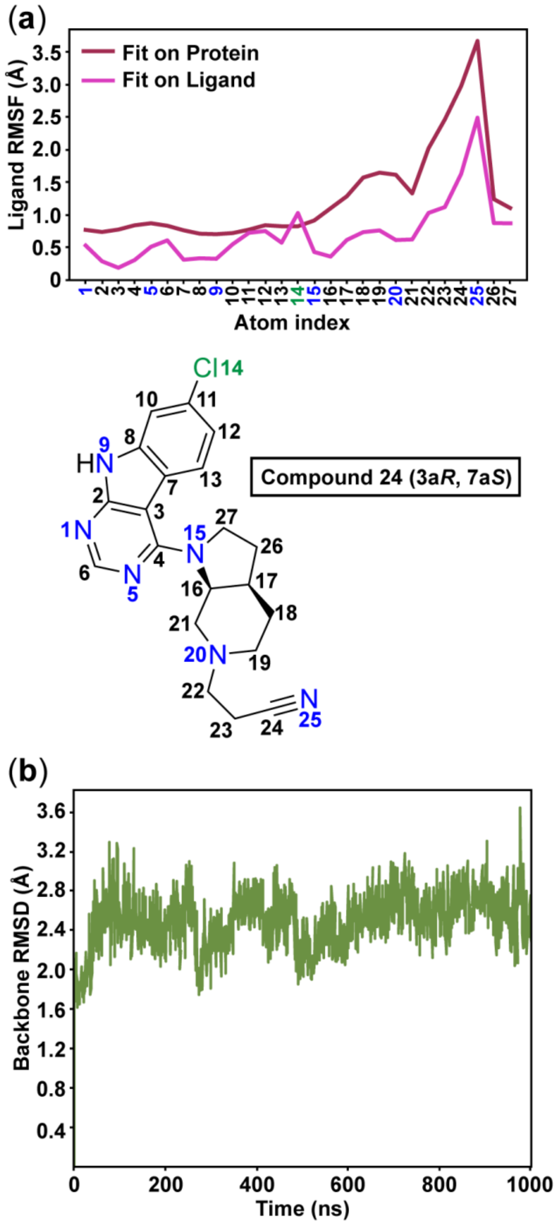

Figure S2. The root-mean-square fluctuation (RMSF) of the ligand **24** (enantiomer (3a*R*, 7a*S*)) (**a**) illustrates a similar flexibility as seen with enantiomer (3a*S*, 7a*R*) (see main text Figure 3b). The root-mean-square deviation (RMSD) of the protein shows that the simulation is stabilized.

ATP Binding Competition of Compound 14b

Compound **14b** was analyzed for its competition with ATP in an ADP Glo GSK-3β assay. To this end, a dilution series of **14b** was incubated with 25 µM, 100 µM and 500 µM of ATP and corresponding IC_{50} values were determined. Results and inhibition curves are listed in Table S1 and Figure S3.

Table S1. IC_{50} values for compound **14b** in the ATP binding competition experiment.

ATP concentration [µM]	GSK-3β IC_{50} [µM] [a]
25	0.764 ± 0.203 [b]
100	2.684
500	9.260

[a] IC_{50} values were determined in an ADP Glo kinase assay, $n = 1$; [b] $n = 5$.

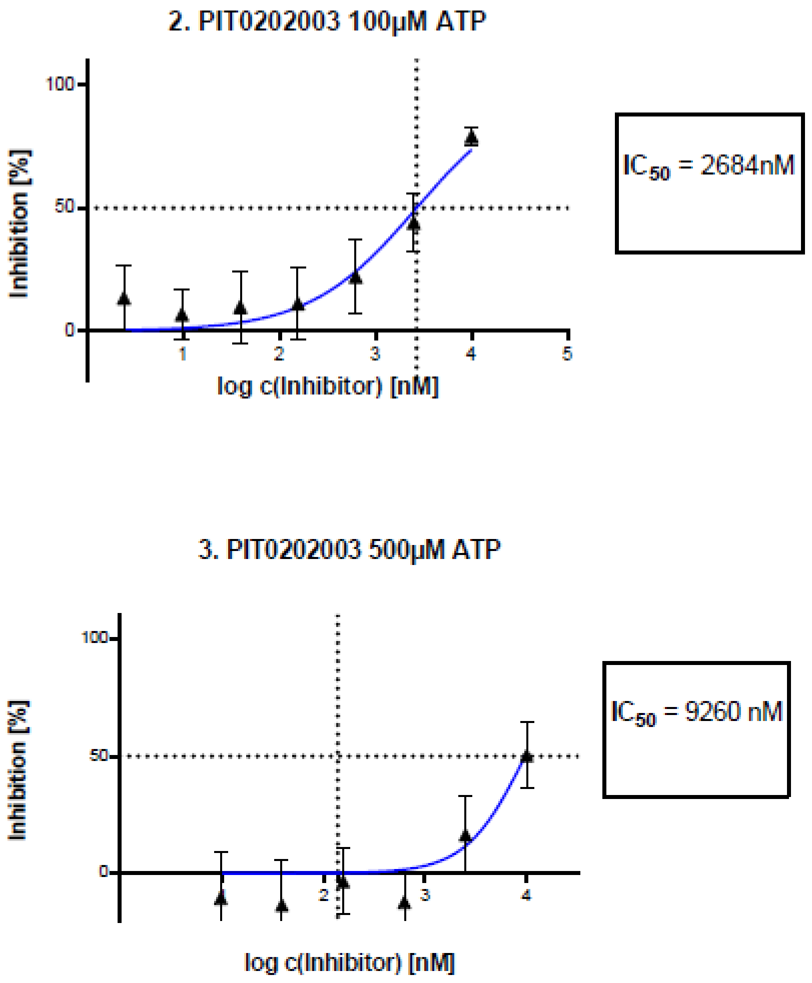

Figure S3. Inhibition curves of compound **14b** in the presence of 100 µM and 500 µM ATP.

JAK3 Inhibition by Compounds 14b and 24

Compound **14b** and **24** were analyzed for their inhibitory activity on JAK3 in an enzyme-linked immunosorbent assay (ELISA). Single point measurements at compound concentrations of 5 μM were conducted. Results are listed in Table S2.

Table S2. JAK3 inhibition of **14b** and **24** in the ELISA.

Cpd.	JAK3 inhibition [%] [a]
**14b**	17.8 ± 6.3
**24**	31.8 ± 3.8

[a] $n = 3$.

Metabolism in Human Liver Microsomes (HLM) of Compounds 14b and 24

Microsomes from liver, pooled from human (male and female) (Lot: SLBQ7487V) were purchased from Merck (Schnelldorf, Germany). The substrate (compounds **14b** and **24**, respectively) (100 µM), an NADPH-regenerating system (5 mM Glucose-6-phosphate, 5 U/mL Glucose-6-phosphate dehydrogenase and 1 mM $NADP^+$) and 4 mM $MgCl_2 \cdot 6\ H_2O$ in 0.1 M Tris buffer (pH 7.4) were preincubated for 5 min at 37°C and 750 rpm on a shaker. The reaction was started by the addition of HLM and then split into aliquots (50 µL). The reaction was quenched at seven time points (0, 10, 20, 30, 60 and 120 min) by addition of 100 µL internal standard (30 µM in MeCN). The samples were vortexed for 30 s and centrifuged (19,800 relative centrifugal force/4°C/10 min). The supernatant was directly used for LC-MS analysis (see below). All incubations were conducted in triplicates. A limit of 1% organic solvent was not exceeded. Propranolol was used as a positive control. Heat inactivated microsomes served as negative control.

The metabolite formation was analyzed with an Alliance 2695 Separations Module (Waters GmbH, Eschborn). The chromatographic separation was performed on a Waters Symmetry C18 column (150 x 4.6 mm; 5 µm) using the gradient listed in Table S3.

Sample temperature: 4°C
Column temperature: 40°C
Injection volume: 10 µL
Flow rate: 0.4 mL/min

Table S3. Chromatographic gradient for separation of metabolism analytes.

Time [min]	**Solvent A [%]** (90% H_2O, 10% MeCN, 0.1% formic acid)	**Solvent B [%]** (MeCN, 0.1% formic acid)
0	90	10
2	90	10
5	50	50
7	50	50
7.01	90	10
13	90	10

The detection was performed on a Micromass Quattro micro triple quadrupole mass spectrometer (Waters GmbH, Eschborn) using the electrospray ionization in the positive-mode.

Spray voltage: 4.5 kV
Desolvation temperature: 250°C
Desolvation gas flow: 600 L/h.

Table S4. Metabolic stability of compounds **14b** and **24**.

Cpd.	Metabolic stability [%] [a]
**14b**	25
**24**	8

[a] Remaining parent compound after an incubation time of 120 min.

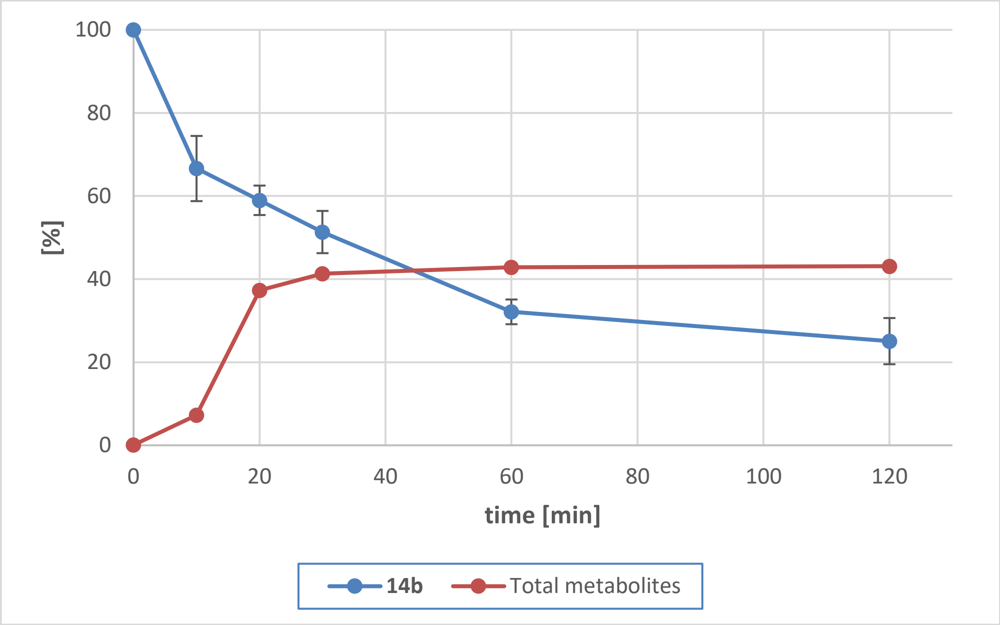

Figure S4. Degradation of compound **14b** during HLM experiment.

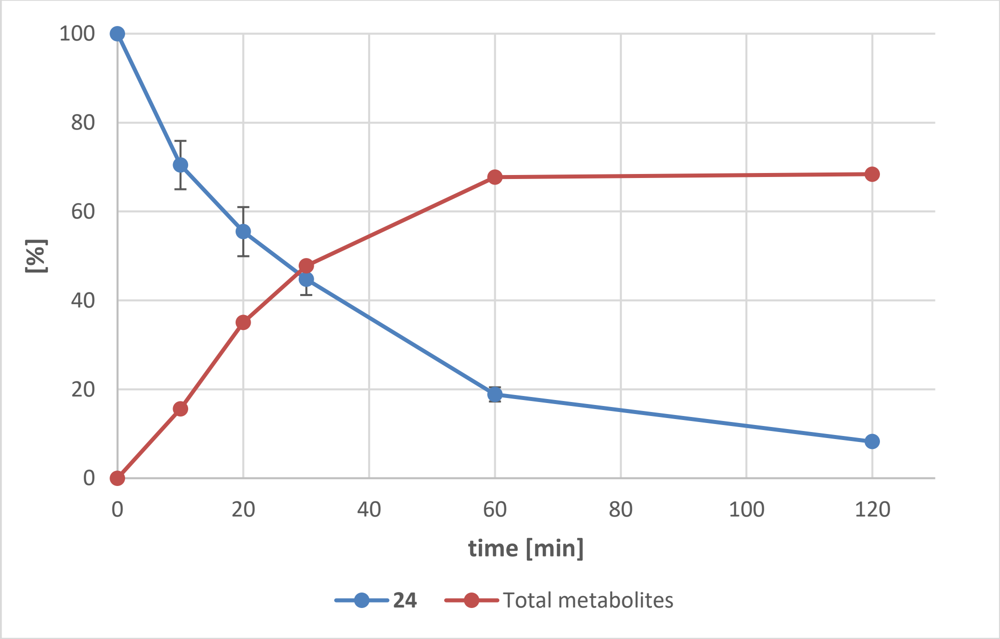

Figure S5. Degradation of compound **24** during HLM experiment.

Structure Determination of Compound 24

Diffraction data were collected at 193 K with a STOE IPDS-2T diffractometer with Mo Kα radiation. Data for atomic coordinates, thermal parameters and reflections can be obtained from the Cambridge Crystallographic Data Centre under the CCDC Nr. 1917242.

Table S5. Data collection and refinement statistics.

Data collection	
Space group	P 2_1/c (monoclinic)
Cell dimensions	determinate from 10604 reflections with 2.6°< θ < 28.4°
a,b,c (Å)	6.7253(5), 17.8510(10), 15.3209(10)Å
β (°)	90.065(5)°
V (Å³), z	1839.3(2), 4
Crystal size (mm³)	0.1 x 0.1 x 0.81 (colorless needle)
Range of Measurement	2° ≤ θ ≤ 28°, -8 ≤ h ≤ 8 -23 ≤ k ≤ 20 -20 ≤ l ≤ 16
No. of reflections:	
Measured	9417
Unique	4356 (R_{int} = 0.0322)
Observed (|F|/σ(F) > 4.0)	3156
**Refinement**	
Nr. of parameters	263
wR2	0.1420
R1(observed), R(all)	0.0534, 0.0872
Goodness of Fit	1.029
Max. deviation of parameters	0.001 * e.s.d.
Max. Peak final	
diff. Fourier synthesis (e *Å⁻³*)	0.22,-0.35
